# Discovery of Novel Oral Protein Synthesis Inhibitors of Mycobacterium tuberculosis That Target Leucyl-tRNA Synthetase

**DOI:** 10.1128/AAC.01339-16

**Published:** 2016-09-23

**Authors:** Andrés Palencia, Xianfeng Li, Wei Bu, Wai Choi, Charles Z. Ding, Eric E. Easom, Lisa Feng, Vincent Hernandez, Paul Houston, Liang Liu, Maliwan Meewan, Manisha Mohan, Fernando L. Rock, Holly Sexton, Suoming Zhang, Yasheen Zhou, Baojie Wan, Yuehong Wang, Scott G. Franzblau, Lisa Woolhiser, Veronica Gruppo, Anne J. Lenaerts, Theresa O'Malley, Tanya Parish, Christopher B. Cooper, M. Gerard Waters, Zhenkun Ma, Thomas R. Ioerger, James C. Sacchettini, Joaquín Rullas, Iñigo Angulo-Barturen, Esther Pérez-Herrán, Alfonso Mendoza, David Barros, Stephen Cusack, Jacob J. Plattner, M. R. K. Alley

**Affiliations:** a European Molecular Biology Laboratory, Grenoble, France; b Anacor Pharmaceuticals, Palo Alto, California, USA; c Institute for Tuberculosis Research, University of Illinois at Chicago, Chicago, Illinois, USA; d Mycobacteria Research Laboratories, Department of Microbiology, Immunology and Pathology, Colorado State University, Fort Collins, Colorado, USA; e TB Discovery Research, Infectious Disease Research Institute, Seattle, Washington, USA; f Global Alliance for TB Drug Development, New York, New York, USA; g Department of Biochemistry and Biophysics, Texas A&M University, College Station, Texas, USA; h Tres Cantos Medicines Development Campus, GlaxoSmithKline, Tres Cantos, Spain

## Abstract

The recent development and spread of extensively drug-resistant and totally drug-resistant resistant (TDR) strains of Mycobacterium tuberculosis highlight the need for new antitubercular drugs. Protein synthesis inhibitors have played an important role in the treatment of tuberculosis (TB) starting with the inclusion of streptomycin in the first combination therapies. Although parenteral aminoglycosides are a key component of therapy for multidrug-resistant TB, the oxazolidinone linezolid is the only orally available protein synthesis inhibitor that is effective against TB. Here, we show that small-molecule inhibitors of aminoacyl-tRNA synthetases (AARSs), which are known to be excellent antibacterial protein synthesis targets, are orally bioavailable and effective against M. tuberculosis in TB mouse infection models. We applied the oxaborole tRNA-trapping (OBORT) mechanism, which was first developed to target fungal cytoplasmic leucyl-tRNA synthetase (LeuRS), to M. tuberculosis LeuRS. X-ray crystallography was used to guide the design of LeuRS inhibitors that have good biochemical potency and excellent whole-cell activity against M. tuberculosis. Importantly, their good oral bioavailability translates into *in vivo* efficacy in both the acute and chronic mouse models of TB with potency comparable to that of the frontline drug isoniazid.

## INTRODUCTION

The aminoacyl-tRNA synthetases (AARSs) are a family of essential enzymes that are required for protein synthesis in all cells (1). Although various family members have been targeted for the design of novel antibacterials (2), only the isoleucyl-tRNA synthetase inhibitor mupirocin is an FDA-approved antibiotic (3). However, mupirocin is approved only for the topical treatment of staphylococcal and streptococcal skin infections (3), and Mycobacterium tuberculosis is naturally resistant to this agent (4). Leucyl-tRNA synthetase (LeuRS) is a class I AARS that has two active sites separated by a distance of 30 Å, a synthetic site that aminoacylates tRNA^Leu^, and an editing site that ensures the fidelity of translation by a proofreading mechanism (5–8). Recently, boron-containing compounds known as oxaboroles have been shown to inhibit LeuRS by the oxaborole tRNA-trapping (OBORT) mechanism (9), which exploits the ability of the boron atom to bond to the *cis*-diols of the 3′-terminal adenosine nucleotide Ade76 of tRNA^Leu^. The resulting covalent adduct traps the 3′ end of tRNA^Leu^ in the editing site in a nonproductive complex, inhibiting leucylation and thereby protein synthesis (9). Here, we report the discovery of novel 3-aminomethyl derivatives that have potent antitubercular activity.

## MATERIALS AND METHODS

### Chemical synthesis.

The syntheses of the compounds are described in detail in the supplemental material.

### Expression, purification, and crystallization of the M. tuberculosis LeuRS editing domain.

A DNA fragment coding for the region spanning G309 to I513 of M. tuberculosis LeuRS (UniProt accession number P67510) was cloned into pETM-11 by using the NcoI and XdeI restriction sites (EMBL). The protein containing an N-terminal six-histidine tag was prepared and purified according to a protocol similar to the one described previously for Escherichia coli LeuRS (8), except that nickel affinity chromatography was conducted at pH 8.0. Protein was stored in buffer comprising 20 mM Tris-HCl (pH 7.4), 100 mM NaCl, 5 mM MgCl_2_, and 5 mM 2-mercaptoethanol. Crystallization was performed at 20°C by the hanging-drop vapor diffusion method. The solutions for the ternary complexes were prepared with 10 mg/ml LeuRS, 5 mM AMP, and 1 mM the corresponding benzoxaborole compound (provided by Anacor Pharmaceuticals, Palo Alto, CA). Initial crystals were obtained at 15 mg/ml LeuRS, 5 mM AMP, and 1 mM the corresponding benzoxaborole compound (provided by Anacor Pharmaceuticals, Palo Alto, CA). Crystals were obtained by mixing 2 μl of this solution with 2 μl of a reservoir solution containing 0.1 M Bis-Tris (pH 5.5), 22% (wt/vol) polyethylene glycol 10000 (PEG 10000), and 0.2 M ammonium acetate. The quality and size of the final diffracting crystal were improved by decreasing the LeuRS concentration to 10 mg/ml and decreasing the PEG 10000 concentration to 17% (wt/vol). The crystals were frozen directly in liquid nitrogen in mother liquor containing 15% (vol/vol) ethylene glycol as a cryoprotectant.

### Structure determination and refinement.

All diffraction data sets were collected at the European Synchrotron Radiation Facility (ESRF, Grenoble, France). Data were integrated and scaled with the XDS suite (10). Further data analysis was performed with the CCP4 suite (11). The structure of the LeuRS:AMP-compound 6 complex was initially solved by molecular replacement with PHASER (12), using the E. coli LeuRS editing domain structure (13) (PDB accession number 2AJG) as a model. The model was improved by automatic building using ARP-wARP (14), and manual adjustments were made with COOT (15). The structures of the complexes with compounds 14 and 16 were solved by using the editing domain of M. tuberculosis LeuRS (described above) as a model. All models were refined by using REFMAC5 with anisotropic *B*-factors. Structure quality was analyzed with MOLPROBITY (16) (http://molprobity.biochem.duke.edu/) and showed all residues in allowed regions (with 95.1 to 98.0% of residues in favored regions) for the different models. Figures were drawn with PYMOL (http://www.pymol.org/).

### Aminoacylation assay.

N-terminal six-histidine-tagged LeuRS from M. tuberculosis H37Rv, which was codon optimized for E. coli (GenScript, Piscataway, NJ, USA), was overexpressed and purified according to the manufacturer's instructions (Novagen, Madison, WI, USA), using an E. coli BL21(DE3) T7 RNA polymerase overexpression strain. Experiments were performed in 96-well microtiter plates, using 80-μl reaction mixtures containing 50 mM HEPES-KOH (pH 8.0), 30 mM MgCl_2_, 30 mM KCl, 13 μM l-[^14^C]leucine (306 mCi/mmol; Perkin-Elmer), 15 μM total E. coli tRNA (Roche, Switzerland), 0.02% (wt/vol) bovine serum albumin (BSA), 1 mM dithiothreitol (DTT), 0.2 pM LeuRS, and 4 mM ATP at 30°C. Reactions were started by the addition of 4 mM ATP to the mixtures. After 7 min, reactions were quenched, and tRNA was precipitated by the addition of 50 μl of 10% (wt/vol) trichloroacetic acid (TCA) and transferred to 96-well nitrocellulose membrane filter plates (Multiscreen HTS, catalog number MSHAN4B50; Millipore). Each well was then washed three times with 100 μl of 5% TCA. Filter plates were then dried under a heat lamp, and the precipitated l-[^14^C]leucine tRNA^Leu^ was quantified by liquid scintillation counting using a Wallac MicroBeta Trilux model 1450 liquid scintillation counter (PerkinElmer, Waltham, MA, USA).

### IC_50_ determination.

To determine the inhibitor concentration that reduces enzyme activity by 50% (IC_50_), increasing concentrations of compound inhibitors that covered the IC_50_ were incubated with LeuRS enzyme, tRNA, and l-leucine for 20 min. Reactions were initiated by the addition of 4 mM ATP to the mixtures. Reactions were stopped after 7 min, and the enzyme was then precipitated and quantified to determine radioactivity. IC_50_ values were determined by using a 4-parameter logistic nonlinear regression model (GraphPad Software Inc., La Jolla, CA, USA).

### Isothermal titration calorimetry experiments.

Isothermal titration calorimetry (ITC) experiments were performed at 25°C by using an ITC200 system (MicroCal; GE Healthcare). The editing domain protein was dialyzed for 12 h against titration buffer (50 mM HEPES-KOH, 30 mM KCl, and 30 mM MgCl_2_ [pH 8.0]) at 4°C. Protein solutions at 50 μM plus AMP at 10 mM in the calorimetric cell were titrated with the appropriate compound dissolved in dialysis buffer. Compound solutions at 1 to 5 mM plus AMP at 10 mM were incubated at 37°C during 1 h before titrations. The heat evolved after each ligand injection was obtained from the integral of the calorimetric signal. The resulting binding isotherms were analyzed by nonlinear least-squares fitting of the experimental data to a single-site model. Analysis of the data was performed by using MicroCal Origin software (OriginLab version 7). Experiments were performed at least twice. The variability in the binding experiments was estimated to be 5% for binding enthalpy and 10% for both the binding affinity and the number of sites.

### Determination of MIC values for M. tuberculosis.

MIC values were determined mainly by using resazurin (alamarBlue) as an indicator for cell growth (17), with additional determinations being performed as described previously by Ollinger et al. (18).

### Selection of Mycobacterium smegmatis ATCC 700084 single-step mutants.

Mutants resistant to compound 1 were isolated on Middlebrook 7H10 medium plus 10% (vol/vol) oleic albumin dextrose catalase (OADC) supplement (Becton Dickinson) containing compound 1 at 4× MIC. Resistance was confirmed by measuring the mutant MIC values essentially as described previously by Collins and Franzblau (17).

### Selection of M. tuberculosis single-step mutants.

M. tuberculosis mutants resistant to compounds 1 and 13 were isolated as described previously by Ioerger et al. (19). Mutants were isolated on Middlebrook 7H10 medium plus 10% (vol/vol) OADC (Becton Dickinson) containing compounds 1 and 13 at 5× or 10× MIC_99_. Resistance was confirmed by measuring the MIC_99_ (20). Genome sequencing and identification of polymorphisms were carried out essentially as described previously by Ioerger et al. (19). In order to determine the rate of spontaneously resistant mutants, M. tuberculosis H37Rv bacteria were grown at 37°C in fresh Middlebrook 7H9 medium-ADC-Tween 80 to mid-exponential phase and then diluted in fresh Middlebrook 7H9 medium-ADC-Tween 80 to 5 × 10^8^ CFU/ml. Middlebrook 7H10-OADC agar plates with 4- and 10-fold MIC values of each compound were inoculated with 10^8^, 10^7^, 10^6^, and 10^5^ CFU/plate, and the plates were incubated at 37°C for 3 to 4 weeks. The frequency of the appearance of resistant mutants was calculated, and isolated colonies were restreaked onto Middlebrook 7H10-OADC agar containing the drugs and onto plates without the drug.

### Time-kill assay.

Compounds were added at 20× MIC to a 10-ml exponential-phase culture of M. tuberculosis H37Rv (∼5 × 10^5^ CFU/ml) in Middlebrook 7H9 medium with 10% (vol/vol) OADC and 0.05% (vol/vol) Tween 80. At the specified time points, aliquots of cultures were withdrawn, serially diluted, and plated onto solid culture medium. Plates were then incubated at 37°C, and CFU were counted after 3 to 4 weeks.

### *In vitro* cytotoxicity assay.

Vero epithelial cells (from African green monkey; ATCC CCL-81) were cultured in Dulbecco's modified Eagle's medium (DMEM) supplemented with 10% fetal bovine serum (FBS) and maintained in a humidified incubator (37°C in 5% CO_2_). Cells were dislodged with a cell scraper, collected by centrifugation, resuspended in fresh medium at ∼10^6^ cells/ml, dispensed into 96-well microtiter plates (100 ml/well), and incubated for 18 h at 37°C. Twofold serial dilutions of test compounds in DMEM with FBS were subsequently added, and cells were incubated for another 72 h. In triplicate studies, the cytopathic effects of compounds were evaluated colorimetrically by using the 3-(4,5-dimethyl-2-thiazolyl)-2,5-diphenyl-2H-tetrazolium bromide (MTT) cell proliferation assay (ATCC). IC_50_ data were obtained from dose-response curves plotted by using GraphPad Prism 5.

### Mitochondrial protein synthesis assay.

The human liver carcinoma-derived HepG2 cell line was obtained from the ATCC (ATCC HB-8065). HepG2 cells were grown in Dulbecco's modified Eagle's medium containing 10% fetal calf serum, 1 mM sodium pyruvate, 0.1 mM essential amino acids, and 50 U/ml penicillin-streptomycin at 37°C with 5% CO_2_. HepG2 cells were seeded into 96-well plates at 3,000 cells/200 μl/well in cell culture medium. Cells were then grown in the presence of the compounds at 37°C in 10% CO_2_ for 7 days in at least duplicate concentrations, with the medium and compounds being replaced on the fourth day. After 7 days, the levels of SDHA (subunit A of the succinate dehydrogenase complex) and COX1 (cytochrome *c* oxidase) were determined by using the MitoSciences In-Cell enzyme-linked immunosorbent assay (ELISA) kit. Janus green staining was used to determined cell viability after 7 days (In-Cell ELISA kit, catalog number MS643).

### Mouse pharmacokinetic analysis.

Mouse pharmacokinetic studies were conducted by using female CD-1 mice for compounds 1, 11, and 12, while BALB/c mice were used for compound 13. Mouse body weights were 19 to 28 g, and on the morning of dosing, mice were split randomly into 3 dosing groups to receive the test article by either intravenous (i.v.) tail vein injection or oral (p.o.) gavage. After dosing, blood samples were collected via cardiac puncture at specific time points (*n* = 3 mice/time point) through 24 h (K_2_EDTA as an anticoagulant) and processed for plasma. Antibiotic concentrations in the plasma samples were analyzed by liquid chromatography-tandem mass spectrometry (LC-MS/MS). The LC-MS/MS analysis was conducted by using analyte/internal standard peak area methods. The internal standard was AN3365 (21), and the instrument used was an API4000 QTRAP instrument (AB Sciex). The limit of quantitation (LOQ) was 1 or 2 ng/ml. Pharmacokinetic analyses of the mean plasma concentration-time profiles were performed by using WinNonlin Pro version 5.2. A compartmental model was used for the data for i.v. dosing, and a noncompartmental model was used for the data for p.o. dosing. The concentration-time curve after an i.v. dose showed a biexponential decline with first-order elimination. Compounds 1 and 13 were formulated in saline (0.9% [wt/vol] NaCl) at 7.5 mg/ml, and the pH was adjusted to >5 by the addition of NaOH. Compound 11 was formulated to 6.5 mg/ml in water-dimethylacetamide-ethanol (EtOH) (76:19:5), and the pH was adjusted to >5 by the addition of NaOH. Compound 12 was formulated to 7.5 mg/ml in PEG 300-propylene glycol (PG)-water (55:25:20), and the pH was adjusted to >5 by the addition of NaOH.

### Mouse plasma protein binding determination.

Compounds were added to 1.5-ml aliquots of mouse plasma at concentrations of 1 and 10 µg/ml and then incubated in a shaking water bath at 37°C for 15 min. Both samples were treated similarly, and a 0.5-ml aliquot was removed from each tube and added to the filter reservoir of Microcon centrifugal filter devices (Ultracel YM-30 with a molecular mass cutoff of 30 kDa). The devices were centrifuged at 1,000 × *g* for 10 min, and 100 μl of the filtrate was transferred to a 96-well plate and diluted 5-fold. Ten-microliter volumes of the samples were injected and analyzed with the LC-MS/MS system. All samples were analyzed in duplicate. Quantitation was based on the peak area ratio of the analyte over the internal standard, and all integrations were performed with peak areas by using Analyst version 1.4.1 (Applied Biosystems, Foster City, CA, USA). Plasma protein binding was calculated based on the following equation:

$$\text{Plasma}\;\text{Protein}\;\text{Binding}\;(\%)=\frac{\left(PeakArea_{Plasma\;Ultrafilrate}^{Spiked}-PeakArea_{Plasma}^{Filtrate}\right)}{PeakArea_{Plasma\;Ultrafiltrate}^{Spiked}}\times 100$$

### Murine model of acute tuberculosis (TB) infection using C57BL/6 GKO IFN-γ mice.

Eight- to 10-week-old female specific-pathogen-free C57BL/6-Ifngtm1ts mice (gamma interferon [IFN-γ] gene-disrupted [gene knockout {GKO}] mice) were purchased from Jackson Laboratories (Bar Harbor, ME). The mice were infected with M. tuberculosis Erdman via a low-dose aerosol exposure in a Middlebrook aerosol generation device (Glas-Col Inc., Terre Haute, IN) as described previously (22). At 1 day postaerosol, three mice were sacrificed to verify the uptake of 50 to 100 CFU of bacteria per mouse. Each treatment group consisted of five mice, and treatment was started at 10 to 13 days postinfection and continued for 9 or 14 consecutive days. Five infected mice were sacrificed at the start of treatment as pretreatment controls. Drugs were administered daily by oral gavage. Lungs were harvested 24 h after the last administration, and all lung lobes were aseptically removed, homogenized, and frozen. Homogenates were plated onto 10% OADC–Middlebrook 7H11 medium for 21 days at 37°C. All animal studies strictly adhered to the protocols and regulations approved by the respective Animal Care and Use Committees of the University of Illinois at Chicago and Colorado State University.

### Murine model of acute and chronic TB infections using C57BL/6J mice.

Specific-pathogen-free 8- to 10-week-old female C57BL/6 mice were purchased from Harlan Laboratories and were allowed to acclimate for 1 week. The experimental design for the acute-infection assay was previously described (23). In brief, for the acute-infection assay, mice were intratracheally infected with 100,000 CFU/mouse (M. tuberculosis H37Rv strain). Products were administered for 8 consecutive days starting 1 day after infection. For the chronic-infection assay, mice were intratracheally infected with 100 CFU/mouse, and the products were administered daily (7 days a week) for 8 consecutive weeks starting 6 weeks after infection. Lungs were harvested 24 h after the last administration. All lung lobes were aseptically removed, homogenized, and frozen. Homogenates were plated onto 10% OADC–Middlebrook 7H11 medium and incubated for 21 days at 37°C. The viable CFU were converted to logarithms, which were then evaluated by one-way analysis of variance, followed by multiple-comparison analysis of variance by a one-way Tukey test (SigmaStat software program). Differences were considered significant at the 95% level of confidence. All animal studies were ethically reviewed and carried out in accordance with European Directive 2010/63/EU and GlaxoSmithKline (GSK) policy on the care, welfare, and treatment of animals.

### Murine model of chronic TB infection using BALB/c mice.

Six- to 8-week-old female specific-pathogen-free immunocompetent BALB/c mice (Charles River, Wilmington, MA) were infected with M. tuberculosis Erdman via low-dose aerosol exposure as described previously (22). At 1 day postaerosol, three mice from each run were sacrificed to verify the uptake of 50 to 100 CFU of bacteria per mouse. Each group consisted of five to six mice at each time point. Treatment was started at 3 weeks postinfection and continued for 12 weeks. Five infected mice were sacrificed at the start of treatment as pretreatment controls. Drugs were administered 5 days per week by oral gavage for 4 weeks. Lungs were harvested 72 h after the last administration. All lung lobes were aseptically removed, homogenized, and frozen. Homogenates were plated onto 10% OADC–Middlebrook 7H11 medium and incubated for 21 days at 37°C. All animal studies strictly adhered to the protocols and regulations approved by the Colorado State University Animal Care and Use Committee.

### Accession numbers.

Atomic coordinates and structure factors for compounds 2, 11, and 13 have been deposited with the Protein Data Bank (PDB) under accession numbers 5AGR, 5AGS, and 5AGT, respectively.

## RESULTS AND DISCUSSION

### Discovery of antitubercular LeuRS inhibitors.

**FIG 1:**
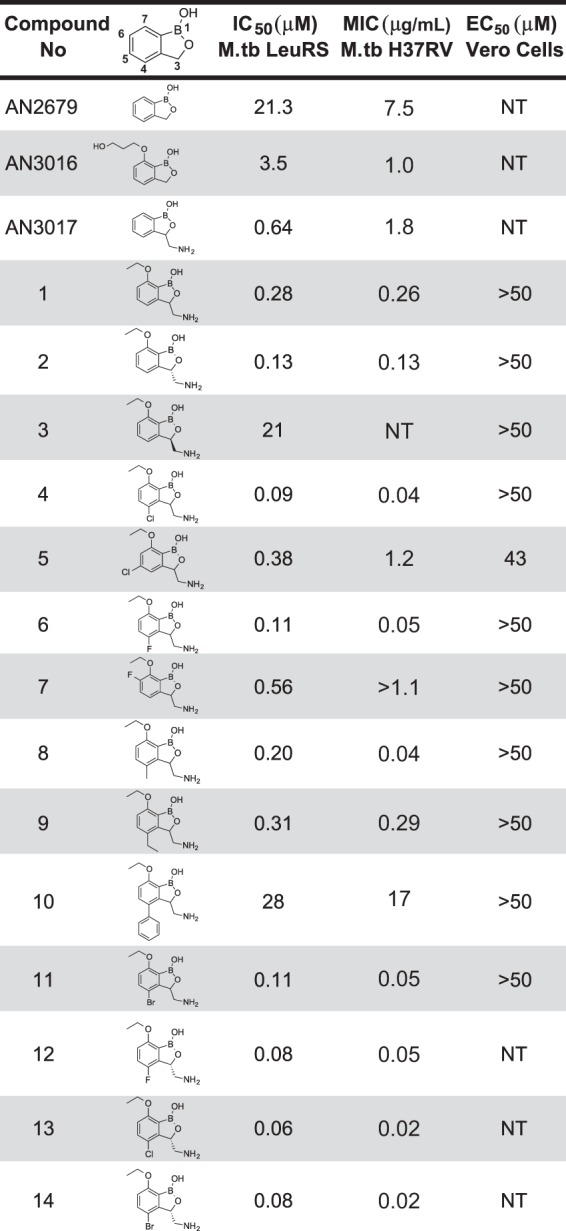
*In vitro* structure-activity relationships. M.tb, M. tuberculosis; NT, not tested.

**FIG 2:**
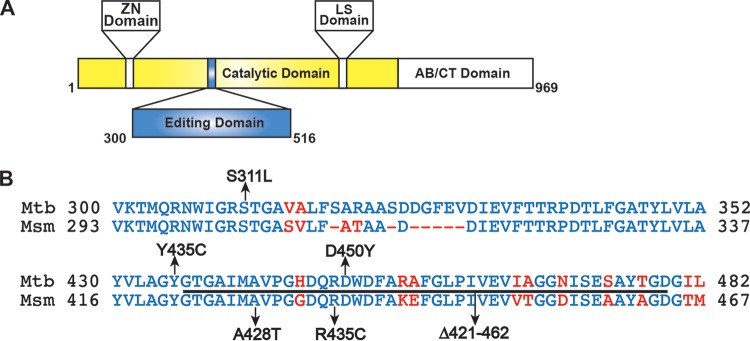
Compound 1-resistant mutants bear mutations in the editing domain of LeuRS. (A) Domain map of M. tuberculosis LeuRS. (B) Amino acid alignment of part of the editing domain of LeuRS from M. tuberculosis and M. smegmatis. Identical residues are shown in blue, and nonidentical residues are shown in red, with arrows indicating where the mutations were found.

**FIG 3:**
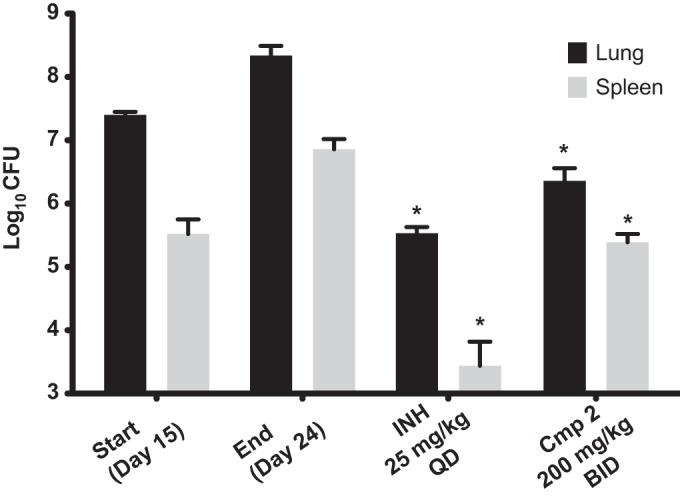
*In vivo* efficacy of compound 2 (Cmp 2) in a murine GKO (C57BL/6-Ifngtm1ts) model of acute TB. Oral treatment was started 15 days (start) after infection with a low-dose aerosol of M. tuberculosis Erdman *lux* and continued for 9 consecutive daily treatments until day 23, when mice were euthanized on day 24 (end). CFU in lungs and spleens were determined, and means from five mice for drug-treated groups and from 6 mice per group for the untreated controls are shown. *, *P* < 0.001 by pairwise multiple-comparison procedures (Tukey test) compared to controls.

A focus library of 20 benzoxaboroles was initially screened against M. tuberculosis H37Rv, which yielded AN3016 and AN3017, with MIC values of 1 μg/ml and 1.8 μg/ml and LeuRS half-maximal inhibitory concentrations (IC_50_) of 3.5 μM and 0.64 μM, respectively (Fig. 1). When we incorporated both the 3-aminomethyl and the 7-ethoxy substitutions into one moiety, compound 1, it gave significantly better activity, with a LeuRS IC_50_ of 0.28 μM and an MIC of 0.26 μg/ml (Fig. 1). To confirm that compound 1 was targeting LeuRS in the cell, we obtained resistant mutants of M. tuberculosis H37Rv and Mycobacterium smegmatis ATCC 700084. The *leuS* gene, which codes for LeuRS, was sequenced from the selected resistant mutants, and mutations were found in both organisms, which was consistent with an OBORT LeuRS inhibitor (Fig. 2 and Table 1). Therefore, we progressed compound 1 into an *in vivo* mouse pharmacokinetic study to determine suitability for testing in a mouse model of acute TB infection. Compound 1 was dosed by oral administration (p.o.) at 30 mg/kg of body weight, which yielded an area under the concentration-time curve from 0 to 24 h (AUC_0–24_) of 15 h · μg/ml, with a maximum concentration in plasma (*C*_max_) of 4.33 μg/ml and oral bioavailability of 55% (data not shown). Since the existing *in vivo* efficacy mouse model used M. tuberculosis Erdman and not M. tuberculosis H37Rv, the MIC of compound 1 was confirmed against the Erdman strain as well as some drug-resistant isolates (Table 2). The M. tuberculosis Erdman MIC value was 0.127 μg/ml, which was not affected by mechanisms of resistance to rifampin, isoniazid (INH), or streptomycin. Compound 1 was then tested in a BALB/c mouse model of acute TB infection at a dose of 100 mg/kg twice a day (BID) for 3 weeks with weekend drug holidays. The control drug PA-824 (pretomanid) (24) was dosed at 100 mg/kg once a day (QD), which gave a 1.9-log_10_ reduction in CFU from mouse lungs, compared to only a 0.4-log_10_ reduction in CFU for compound 1 (data not shown). Therefore, we separated the two enantiomers in compound 1 by chiral high-performance liquid chromatography (HPLC) and determined their activities (Fig. 1). The active enantiomer was the (*S*)-isomer, compound 2, which had a LeuRS IC_50_ of 0.13 μM and an MIC of 0.13 μg/ml, while the (*R*)-isomer was barely active, with an IC_50_ of 21 μM. The active enantiomer compound 2 was then tested at 200 mg/kg BID for 9 days in the model of acute TB infection by using an IFN-γ gene knockout (GKO) mouse (25), which showed a 2-log_10_ reduction in lung CFU and a 1.5-log_10_ reduction in spleen CFU compared to control mice (Fig. 3). However, this was not as good as the frontline drug INH, which gave 2.8- and 3.4-log_10_ reductions in CFU from lungs and spleen, respectively, when dosed at 25 mg/kg.

**TABLE 1:** MIC values for compound 1-resistant mutants^*a*^

Organism	MIC (μg/ml)^*b*^
M. tuberculosis	
H37Rv	0.6–1.3
RM1*leuS* Y435C	21
RM2*leuS* S311L	21
RM3*leuS* D450Y	21
RM4*leuS* S311L	21
M. smegmatis	
ATCC 700084	1
RM1*leuS* Δ421–462	>256
RM2*leuS* A428T	64
RM3*leuS* R435C	8
RM4*leuS* A428T	64

a Residues Y435 and D450 stabilize the adduct formed by compound 1 with AMP in the editing site of M. tuberculosis LeuRS (Fig. 4B). Residue S311, like the equivalent E. coli LeuRS residue (13), interacts with the phosphate of Ade76, thus stabilizing the adduct in the editing site. However, S311 is located at the flexible N-terminal part of our editing domain construct of M. tuberculosis LeuRS and thus is not visible in the crystal structure (Fig. 4B). RM, resistant mutant.

b MIC values for M. tuberculosis were determined on agar, and those for M. smegmatis were determined in liquid cultures.

**TABLE 2:** Compound 1 MIC values against M. tuberculosis Erdman and monoresistant isolates compared with known standards^*a*^

Strain	MIC (μg/ml)				
	Compound 1	PA-824	RIF	INH	STR
M. tuberculosis Erdman	0.127	0.116	0.018	0.244	0.369
RIF^r^	0.120	0.128	>4	0.383	0.216
INH^r^	0.059	≤0.063	0.037	>8	0.202
STR^r^	0.113	0.189	0.082	0.344	>16

a RIF, rifampin; INH, isoniazid; STR, streptomycin; RIF^r^, strain with resistance to rifampin.

### M. tuberculosis LeuRS inhibitor cocrystallization.

In order to improve potency, we performed structural and biophysical studies to understand the mode of binding of these novel 3-aminomethyl benzoxaboroles to M. tuberculosis LeuRS. Crystallization trials with different editing domain constructs of M. tuberculosis LeuRS were attempted in the presence of compound 2 with either AMP or longer nucleotides such as CytAde or CytCytAde, which might act as surrogates for the 3′ end of the tRNA acceptor stem (26). An editing domain construct encompassing residues G309 to I513 gave cocrystals with compound 2 and AMP that diffracted to a 1.3-Å resolution, which permitted structure determination (Fig. 4A and B; see also Table S1 in the supplemental material). Compound 2 forms a bidentate covalent adduct with AMP (Fig. 4B), which mimics Ade76 of the tRNA acceptor end (7, 8, 21). Amino acid residues T336 to T337 of the threonine-rich region provide multiple H-bonding interactions to the covalent adduct, and L432 and Y435 of the AMP binding loop have extensive H-bonding and hydrophobic contacts with AMP (Fig. 4B). In addition, the amino group of compound 2 makes three key interactions with the carboxylic acid side chains of D447 and D450 and the carbonyl group of M441. The 7-ethoxy substitution not only enables a new interaction with R449 but also packs with the Ade76 ribose, thus further stabilizing the boron-tRNA adduct (Fig. 4B). Superposition of the compound 2 adduct-bound structure with that of the E. coli LeuRS editing domain with methionine bound (13) shows that the 3-aminomethyl benzoxaborole moiety occupies the same position as the noncognate amino acid (Fig. 4C). Although this moiety mimics the interactions established by the amino and the oxygen carbonyl groups of methionine, it lacks atoms at the positions of the S^δ^-C^ε^ atoms of methionine (Fig. 4C), which suggests that there is additional space to make further interactions.

**FIG 4:**
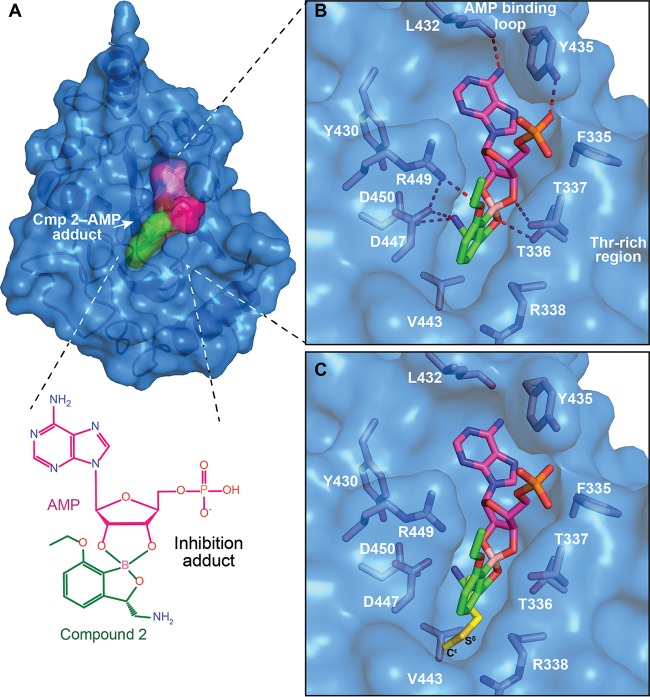
X-ray cocrystal structure of LeuRS with compound 2. (A) Crystal structure of the M. tuberculosis LeuRS editing domain in complex with compound 2 (carbon atoms are shown in green)-AMP (carbon atoms are shown in magenta). Color coding is the same throughout all figures, with blue for nitrogen, red for oxygen, pink for boron, orange for phosphorus, and yellow for sulfur. (B) Zoomed view into the editing site of M. tuberculosis LeuRS showing the compound 2-AMP adduct and the key residues establishing important hydrogen bonds (red dashed lines), with only the H bond from the 3-aminomethyl to M441 being omitted for clarity. (C) Overlay of the LeuRS editing domain of M. tuberculosis and E. coli in complex with methionine (in yellow) (PDB accession number 2AJH). The 3-aminomethyl group of compound 2 mimics the amino group of methionine, including the interaction with the bacterium-specific residue D447.

### Structure-activity relationship of potent antitubercular agents.

Several derivatives were synthesized with different substitutions at position 4 as well as at positions 5 and 6 to explore this hypothesis (Fig. 1). The halogen substitutions 5-Cl (compound 5) and 6-F (compound 7) were not well tolerated, with LeuRS activity being worse than the original compound 1 and MIC values of 1.1 μg/ml or greater. The most potent analogs were compounds with halogen substitutions at position 4, bromo (compound 11), chloro (compound 4), and fluoro (compound 6) substitutions, which improved MIC values more than 5-fold over those compound 1 (Fig. 1). The phenyl (compound 10) substitution was not tolerated, with a LeuRS IC_50_ of 28 μM (Fig. 1). However, it must be noted that the significant improvements in MIC values for compounds 4, 6, and 11 were not fully reflected in their IC_50_ values as determined by using an aminoacylation assay with M. tuberculosis LeuRS, which could be due to the way in which OBORT inhibitors indirectly inhibit aminoacylation by preventing binding of Ade76 to the aminoacylation active site (9). We therefore decided to measure the direct binding of the compounds to the editing domain using isothermal titration calorimetry (ITC) and found that the 4-Cl and 4-Br substitutions significantly enhanced the affinity of the compounds for the M. tuberculosis LeuRS editing domain (Table 3). This increased affinity is due to a significant gain in the enthalpic contribution (3.1 to 4.4 kcal mol^−1^), which is consistent with additional favorable interactions being established by the halogen atoms in the editing site. To confirm that whole-cell activity was derived from the inhibition of LeuRS, we selected 6 M. tuberculosis mutants resistant to compound 13 and sequenced their *leuS* genes, while two additional mutants were selected for whole-genome sequencing. All 8 resistant mutants had a single nucleotide polymorphism (SNP) in their *leuS* genes, and the mutations were located in the editing domain, as expected for OBORT LeuRS inhibitors (9, 21, 27) (see Table S2 in the supplemental material). To further explore interactions at the 4-position, we cocrystallized compounds with 4-Cl and 4-Br substitutions in the presence of AMP and solved the structures of the ternary complexes at 1.45- and 1.47-Å resolutions, respectively (see Table S1 and Fig. S1 in the supplemental material). The structures showed that the halogenated compounds bind to the editing site without major structural changes, and as predicted, the 4-Cl/Br atoms now occupied the position of the sulfur in bound methionine (see Fig. S2A in the supplemental material), allowing van der Waals interactions with the neighboring protein atoms (see Fig. S2B in the supplemental material). These results confirmed the importance of the size and nature of the substitution at position 4 and agreed well with the *in vitro* activities and thermodynamic analyses (see Fig. S2C in the supplemental material).

**TABLE 3:** Thermodynamic analysis of interactions between M. tuberculosis LeuRS and benzoxaborole compounds^*a*^

Compound	*Kd* (μM)	Δ*G*_ap_ (kcal mol^−1^)	Δ*H*_ap_ (kcal mol^−1^)	−*T*Δ*S*_ap_ (kcal mol^−1^)	No. of sites
2	3.7	−7.4	−1.1	−6.3	1.05
13	0.075	−9.7	−4.2	−5.5	1.19
11	0.040	−10.0	−5.5	−4.6	1.02

a The errors in the thermodynamic binding parameters are ∼5% for the apparent binding enthalpy and 10% for the apparent binding constant and the number of sites. *Kd*, dissociation constant; Δ*G*, change in Gibb's free energy; Δ*H*, change in enthalpy; Δ*S*, change in entropy. Values are the averages of data from at least 2 independent experiments.

**FIG 5:**
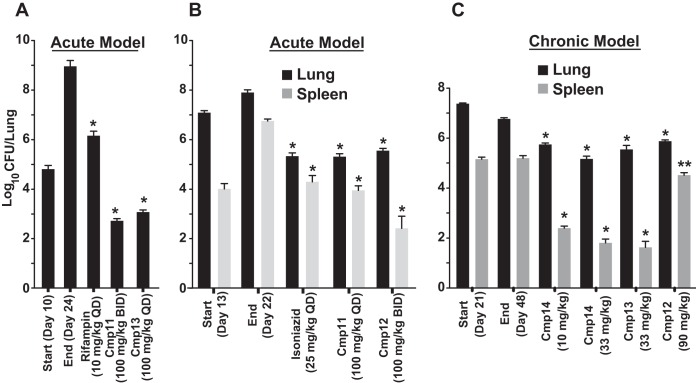
*In vivo* efficacy of compounds 11, 12, 13, and 14 in models of acute and chronic of TB infections. (A) *In vivo* efficacy in a murine GKO (C57BL/6-Ifngtm1ts) model of acute TB. Compounds were dosed orally daily for 14 days after 10 days of infection (start) with a low-dose aerosol of M. tuberculosis Erdman. Mean lung CFU were determined from five mice at the end. (B) *In vivo* efficacy in a murine GKO (C57BL/6-Ifngtm1ts) model of acute TB. Oral treatment was started 13 days after infection (start) with a low-dose aerosol of M. tuberculosis Erdman *lux* and continued for 9 consecutive daily treatments until day 21, when mice were sacrificed on day 22 (end). Mean lung CFU were determined from five mice at the end. (C) *In vivo* efficacy in a murine BALB/c model of chronic TB infection. Compounds were dosed orally 5 days a week for 4 weeks after infection with M. tuberculosis Erdman with a low-dose aerosol 21 days prior (start). Lung and spleen CFU were determined from six mice at the end. **, *P* < 0.01; *, *P* < 0.001 (by pairwise multiple-comparison procedures [Tukey test] compared to controls).

We selected the three most potent compounds, compounds 11, 12, and 13, for *in vivo* murine pharmacokinetic analysis, and we dosed mice both i.v. and p.o. All three compounds showed improvements in plasma exposure as measured by AUC after oral administration over compound 2 (Table 4). Therefore, we tested them in a GKO mouse model of acute TB, which showed that all three compounds were very efficacious, with racemate compound 11 having efficacy similar to that of isoniazid (Fig. 5A and B). In a BALB/c mouse model of chronic TB infection, all compounds showed good efficacy (Fig. 5C), with compound 14, the (*S*)-isomer of compound 11, being the most potent. In addition, we observed that compounds 13 and 14 did not show any cross-resistance against multidrug-resistant isolates (see Table S3 in the supplemental material).

**TABLE 4:** Murine pharmacokinetic parameters

Parameter^*a*^	Value for compound:				
	2	11	12	13	14
i.v.^*b*^					
Dose (mg/kg)	30	15	30	30	30
*C*_max_ (μg/ml) at 5 min	8.9	18.0	13.7	13.6	17.1
CL (ml/h/kg)	2,180	328	1,119	582	687
*V*_ss_ (ml/kg)	2,116	968	3,805	3,142	3,221
MRT (h)	2.1	3.0	3.4	5.4	4.7
AUC_0–∞_ (h · μg/ml)	13.8	45.8	26.8	51.6	43.7
α-*t*_1/2_ (h) (% AUC)	0.06 (5)	0.09 (2)	0.11 (7)	0.10 (2)	0.05 (5)
β-*t*_1/2_ (h) (% AUC)	1.5 (95)	2.08 (98)	2.53 (93)	3.83 (98)	3.40 (95)
p.o.^*c*^					
Dose (mg/kg)	30	30	30	30	30
*C*_max_ (μg/ml)	3.4	7.2	5.0	6.4	6.3
*T*_max_ (h)	0.50	1.00	1.00	0.25	0.50
AUC_0–24_ (h · μg/ml)	13.2	35.9	23.8	47.5	57.6
Terminal *t*_1/2_ (h)	1.8	2.7	2.7	3.1	3.6
Bioavailability (%)	96	39	89	92	100
Mouse PPB (%)	6	50	16	23	

a CL, clearance; *V*_ss_, volume of distribution at steady state; MRT, mean residence time; *t*_1/2_, half-life; *T*_max_, time to maximum concentration of drug in serum. PPB, plasma protein binding.

b WinNonlin two-compartment analysis with iterative weighting.

c WinNonlin noncompartmental analysis with uniform weighting.

### Inhibition of mitochondrial protein synthesis.

Although protein synthesis inhibitors are validated TB drugs, they are associated with some safety concerns, for example, myelosuppression and neuropathy observed with linezolid (28) and deafness induced by aminoglycosides (29). The similarity between the bacterial and mitochondrial protein synthesis machineries (30) and their subsequent inhibition of mitochondrial protein synthesis is thought to drive these toxicities. Therefore, we tested the ability of compound 14 and some close analogs to inhibit mitochondrial protein synthesis in the human liver carcinoma cell line HepG2 (Table 5). The ribosomal protein synthesis inhibitors linezolid, chloramphenicol, and doxycycline inhibited the synthesis of the mitochondrially derived COX1 protein with 50% effective concentrations (EC_50_) of between 23 and 31 μM, while erythromycin and compounds 12, 13, and 14 had EC_50_ of >150 μM. Although this could be due to poor mitochondrial penetration, it is interesting to note that human mitochondrial LeuRS is known to be editing defective, as it lacks key conserved amino acid residues in the AMP binding loop and amine binding pocket (31).

**TABLE 5:** Mitochondrial protein synthesis inhibition^*a*^

Compound	Mean EC_50_ (μM) ± SD		
	COX1	SDHA	Cell viability
Compound 12	>150	39.5 ± 9.2	23.0 ± 1.4
Compound 13	>150	20.5 ± 2.1	80.0 ± 5.7
Compound 14	>150	21.5 ± 6.4	106 ± 37.5
Linezolid	27.3 ± 10.8	>150	>150
Chloramphenicol	31.4 ± 23.2	>150	110 ± 14.1
Doxycycline	23.7 ± 6.4	109 ± 29	118 ± 35.5
Erythromycin	>150	>150	>150

a COX1 is cytochrome *c* oxidase, which is a mitochondrial protein that is synthesized by mitochondrial ribosomes. SDHA is subunit A of the succinate dehydrogenase complex, which is a mitochondrial protein that is synthesized by cytoplasmic ribosomes. Janus green staining was used to determine cell viability after 7 days.

### *In vitro* and *in vivo* activities of compound 14.

**FIG 6:**
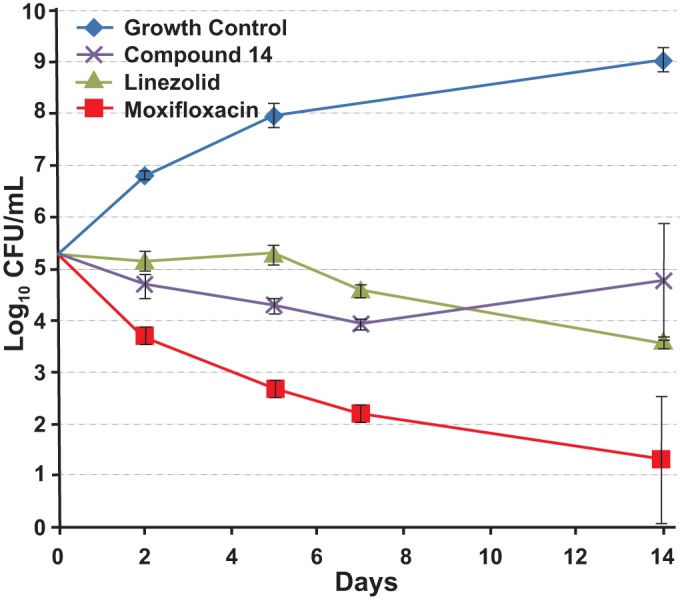
M. tuberculosis H37Rv *in vitro* kill kinetics. Cells were incubated with compounds at 20 times their MIC values for different times over 14 days in 10 ml of Middlebrook 7H9 medium containing 10% (vol/vol) ADC and 0.05% (vol/vol) Tween 80. The MIC values used in this experiment were 0.013 μg/ml, 0.6 μg/ml, and 0.06 μg/ml for compound 14, linezolid, and moxifloxacin, respectively. The means and the standard deviations of data from triplicate cultures at each point are shown.

**FIG 7:**
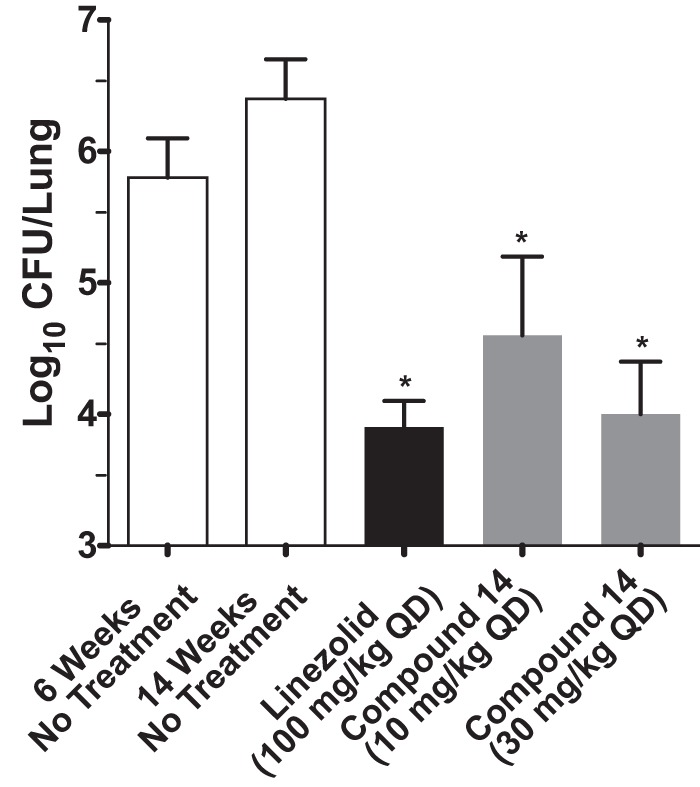
Efficacy of compound 14 in a mouse model of chronic TB infection. C57BL/6J mice were infected with M. tuberculosis H37Rv intratracheally (∼10^2^ CFU) and were dosed once daily for 8 weeks starting 6 weeks after infection. Mice were sacrificed 24 h after the last drug administration. Every column represents the mean values ± standard deviations of data from 7 mice per group for untreated and linezolid-treated groups and from 3 mice for compound 14-treated mice. *, *P* < 0.001 by pairwise multiple-comparison procedures (Tukey test) compared to controls.

**FIG 8:**
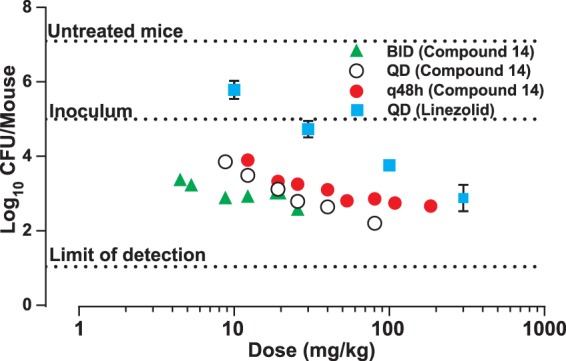
Efficacy of compound 14 in a mouse model of acute TB infection under different dosing regimes of once a day (QD), twice a day (BID), or every other day (q48h). C57BL/6J mice were infected with M. tuberculosis H37Rv intratracheally (∼10^5^ CFU) and were dosed starting on the following day after infection for 8 days. Only one dose was administered on day 8 under the BID schedule. Mice were sacrificed at least 24 h after the last drug administration. Every dot represents one mouse data point except for linezolid (mean of data for 5 mice ± standard deviation).

Since racemate compound 11 had activity similar to that of isoniazid, which is an *in vitro* bactericidal compound (32), in the GKO mouse model of acute infection (Fig. 5B), we tested the pure enantiomer compound 14 for *in vitro* bactericidal activity over 14 days at 20 times its MIC (Fig. 6). The profile for compound 14 was very similar to that of the bacteriostatic protein synthesis inhibitor linezolid, which was different from that of moxifloxacin, a known bactericidal compound (33). Therefore, further tests of compound 14 in a murine chronic TB infection model (Fig. 7) were performed in parallel with the protein synthesis inhibitor linezolid. Compound 14 at 30 mg/kg QD showed good efficacy, resulting in a 2.4-log_10_ reduction in CFU, compared with a 2.6-log_10_ reduction in CFU for 100 mg/kg of linezolid QD. In order to establish the optimal dosing regimen for compound 14, we tested it in the murine model of acute TB infection and compared efficacies using dosing regimes of BID, QD, and every 48 h (q48h) (Fig. 8). Similar to results with the chronic infection model, compound 14 was more active at lower doses than linezolid, and q48h dosing was as efficacious as QD or even BID dosing, which suggests that the preliminary pharmacodynamic driver for efficacy was the AUC/MIC ratio.

### Resistance and LeuRS inhibitors.

Emergence of resistance during streptomycin monotherapy (34) and its noted reduction by the addition of *p*-aminosalicylic acid (35, 36) lead to the paradigm of combination TB drug therapy. The current core TB regimen calls for a four-drug combination of isoniazid, rifampin, ethambutol, and pyrazinamide. Although compound 14 has a lower frequency of *in vitro* resistance than isoniazid (Table 6), the emergence of resistance to epetraborole (GSK2251052/AN3365), another 3-aminomethylbenzoxaborole LeuRS inhibitor, in a minority of patients in a complicated urinary tract infection trial might suggest some caution (27). However, the addition of trimethoprim to rifampin, which has a similar resistance problem, in a urinary tract infection trial demonstrated the benefit of combination therapy in overcoming the emergence of rifampin-resistant strains (37). This suggests that the risk from the emergence of resistance to OBORT LeuRS inhibitors will likely be mitigated when used in combination therapy.

**TABLE 6:** *In vitro* frequencies of resistance^*a*^

Compound	Resistance frequency at:	
	4× MIC	10× MIC
Compound 14	4.6 × 10^−6^	3.9 × 10^−6^
Isoniazid	ND	1.8 × 10^−5^
Moxifloxacin	1.7 × 10^−7^	1.1 × 10^−8^

a The MIC values for compound 14, isoniazid, and moxifloxacin on Middlebrook 7H10 agar were determined to be 0.2, 0.06, and 0.08 μg/ml, respectively. ND, not determined.

### Beneficial properties.

Since combination therapy necessitates a larger armamentarium than regular monotherapy, the demonstration for the first time that an oral AARS inhibitor can be a potent antitubercular agent adds a potential new tool to fight TB, which is timely noting the recent onset of totally drug-resistant (TDR) TB. In addition, the combination of low plasma protein binding, molecular weight (207 to 285), and logD_7.4_ (−0.04 to 0.76) values, like the frontline TB drugs isoniazid, pyrazinamide, and ethambutol, suggests that this novel chemical class deserves further optimization and, hopefully, progression into clinical trials.

## Supplementary Material

Supplemental material
