# Supplementary material for: Discovery of Novel Oral Protein Synthesis Inhibitors of Mycobacterium tuberculosis That Target Leucyl-tRNA Synthetase
Source: Antimicrob Agents Chemother. 2016 Sep 23;60(10):6271–80. doi: 10.1128/AAC.01339-16 (PMC5038265; doi:10.1128/AAC.01339-16)
Supplement: Supplemental material [file supp_60_10_6271__index.html]

Discovery of Novel Oral Protein Synthesis Inhibitors of Mycobacterium tuberculosis That Target Leucyl-tRNA Synthetase — Supplemental material 

# Discovery of Novel Oral Protein Synthesis Inhibitors of Mycobacterium tuberculosis That Target Leucyl-tRNA Synthetase

## Supplemental material

- Supplemental file 1 -

  Supplemental material: chemical synthetic methods, Tables S1 to S3, and Figures S1 and S2.

  PDF, 5.5M
