# Supplementary material for: Discovery of Novel Oral Protein Synthesis Inhibitors of Mycobacterium tuberculosis That Target Leucyl-tRNA Synthetase
Source: Antimicrob Agents Chemother. 2016 Sep 23;60(10):6271–80. doi: 10.1128/AAC.01339-16 (PMC5038265; doi:10.1128/AAC.01339-16)
Supplement: Supplemental material [file AAC.01339-16_zac010165598so1.pdf]

CHEMICAL SYNTHETIC METHODS

Starting materials used were either commercially available or prepared according to literature procedures and had experimental data in accordance with those reported. The 5-, 6- and 7-substituted 3-aminomethyl benzoxaboroles were synthesized from appropriate starting materials according to a modified procedure reported by Hernandez (2). The C-4 chloro or bromo analogs were prepared by chlorination or bromination of C-4 unsubstituted compounds. The C-4 alkyl or aryl derivatives were made by palladium-catalyzed coupling reaction of 4-bromo compounds with organotin reagents. Yields refer to purified products and are not optimized. The purity of all tested compounds was >95% by HPLC. Enantiomers were obtained by chiral separation of the racemic mixture. The enantiomeric excess (ee) of all tested enantiomers was >98%. ^{1}H NMR spectra were recorded on a Varian or Bruker 400 MHz spectrometer. Chemical shifts were reported in ppm and were referenced to the appropriate residual solvent signals, such as 2.49 ppm for DMSO-d_6 and 7.26 ppm for $CDCl_3$. LC-MS data were obtained using an Agilent LC-MS 1200 with 6110 MS detector. The mass spectrometer was equipped with an electrospray ion source (ESI) operated in a positive or negative mode. Flash column chromatography was typically preformed using silica gel 60 (230-400 mesh). Typical solvents used for flash chromatography or thin layer chromatography (TLC) were mixtures of DCM/MeOH, and hexane/EtOAc. HPLC analysis was performed on a Shimadzu HPLC system or a Water 600 Controller system using either of the following columns; a Venusil XBP-C18 (50 × 4.6 mm I.D.), a Shimpack VP-ODS (150 × 4.6 mm I.D.), or an UPLC BEH C18 (50 × 2.1 mm I.D.) column. Purification using prep-HPLC was accomplished using Shimadzu LC-8A System or a Waters Prep LC 4000 System using either of the following columns; a Phenomenex Luna C18 (300 × 50 mm I.D.), a Dasio C18 (250 × 50 mm I.D.) or a Waters X-Terra Prep C18 (100 × 30 mm I.D.) column. Typically, the mobile phase used for HPLC was a linear gradient of water (A) and acetonitrile (B). The water and acetonitrile were mixed with 0.05% or 0.025% TFA. The eluent were monitored with UV detector at 220. Chiral separation was performed on a Thar preparative SFC 80 system using either a Chiralpak AD (200 × 25 mm I.D., 20 µM) or a ChiralCel OZ-H (250 × 30 mm I.D.) column and eluted with a carbon dioxide and methanol mobile phase. The flow rate was maintained at 65-70 mL/min, and the eluent was monitored with an UV detector at 220 nm. For enantiomeric excess determination, chiral HPLC analysis was performed on a Thar analytical SFC system with a Chiralpak AD-H (150 × 4.6 mm I.D.) or a

ChiralCel OZ-H (150 × 4.6 mm I.D.) column and eluted with a carbon dioxide and methanol mobile phase at a flow rate of approximately 2.4-4 mL/min.

Scheme 1

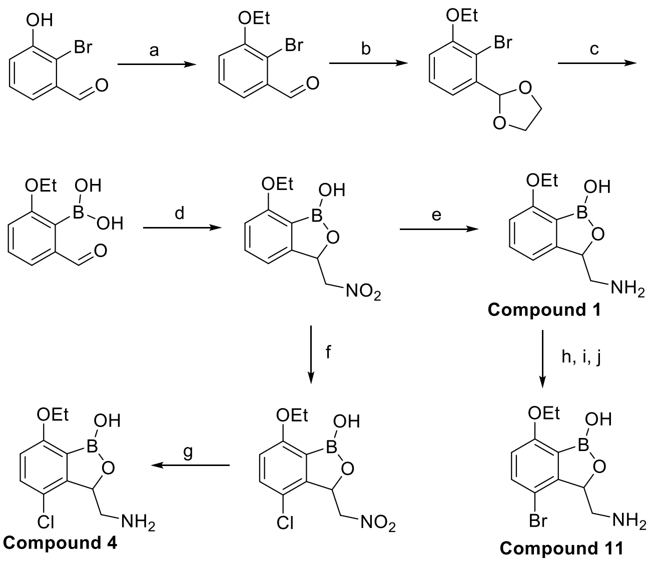

Reagents and conditions: (a) K_2CO_3, DMF, 70 ºC; (b) $HOCH_2CH_2OH$, TsOH, toluene, reflux; (c) (1) *n*-BuLi, (*i*PrO)$_3$B, THF, -78 ºC; (2) 2 N HCl, rt; (d) CH_3NO_2, NaOH, CTAB, water/THF; (e) Raney Ni, H_2, NH_3/EtOH, then HCl/EtOH; (f) NCS, 80 ºC, DMF; (g) Raney Ni, H_2, NH_3/EtOH, then HCl/Et_2O; (h) di-*tert*-butyl dicarbonate, TEA, DCM, 0 ºC; (i) NBS, CH_3CN, 90 ºC; (j) TFA, DCM.

3-(Aminomethyl)-7-ethoxybenzo[c][1,2]oxaborol-1(3H)-ol hydrochloride (Compound 1)

A mixture of 2-bromo-3-hydroxybenzaldehyde (163 g, 0.81 mol), K_2CO_3 (123 g, 0.89 mol) and bromoethane (132 g, 1.22 mol) in DMF (1200 mL) was stirred at 70 °C for 2 h. The reaction was quenched with water, and the resulting mixture was extracted with EtOAc. The combined organic layers were washed with water and brine, dried over anhydrous Na_2SO_4, and concentrated *in vacuo*. The residue was purified by silica gel chromatography eluted with hexane/ethyl acetate to give 2-bromo-3-ethoxybenzaldehyde (157 g, yield 85%). ^{1}H NMR (400 MHz, $CDCl_3$) δ 10.45 (s, 1H), 7.50-7.52 (t, 1H), 7.34-7.38 (m, 1H), 7.11-7.13 (m, 1H), 4.13-4.19 (q, 2H), 1.51-1.54 (t, 3H). To a solution of 2-bromo-3-ethoxybenzaldehyde (157 g, 0.69 mol) and glycol (340 g, 5.50 mol) in toluene (600 mL) was added *p*-toluenesulfonic acid (13 g, 0.07 mol). The reaction flask had a Dean-Stark condenser attached and the reaction mixture was refluxed to remove water for 4 h. The mixture was then cooled to room temperature and concentrated *in vacuo*. The residue was purified by silica gel chromatography eluted with hexane/ethyl acetate to give 2-(2-bromo-3-ethoxyphenyl)-1,3-dioxolane (149 g, yield 79%). ^{1}H NMR (400 MHz, $CDCl_3$) δ 7.33-7.37 (m, 1H), 7.14-7.16

(m, 1H), 7.10-7.14 (m, 1H), 5.99 (s, 1H), 4.05-4.14 (m, 6H), 1.32-1.38 (t, 3H). To a solution of 2-(2-bromo-3-ethoxyphenyl)-1,3-dioxolane (147 g, 0.54 mol) in anhydrous THF (1000 mL) was added *n*-BuLi (2.5 M in THF, 432 mL, 1.08 mol) dropwise at -78 ^{o}C under N_2. After stirring at -78 ^{o}C for 2 h, triisopropyl borate (201 g, 1.08 mol) was added dropwise. The mixture was stirred at -78 ^{o}C for 4 h. After the reaction was quenched by adding saturated solution of NH_4Cl (300 mL), the resulting mixture was extracted with EtOAc (3 × 500 mL). The combined organic layers were washed with water (300 mL) and brine (300 mL), dried over anhydrous Na_2SO_4, and concentrated *in vacuo* to give diisopropyl 2-(1,3-dioxolan-2-yl)-6-ethoxyphenylboronate without further purification (150 g, 86% yield). To a solution of this crude compound (150 g, 0.47 mol) in THF (500 mL) was added 2 N HCl (200 mL) slowly. After being stirred for 1.5 h, the reaction mixture was basified with 20% NaOH solution to pH 12 and then washed with EtOAc (2 × 200 mL). The aqueous layer was acidified by using 2 N HCl to pH 2 and extracted with EtOAc (3 × 200 mL). The combined organic layers were dried over anhydrous Na_2SO_4 and concentrated *in vacuo* to give (2-ethoxy-6-formylphenyl)boronic acid as an oil without further purification (83 g, yield 91%).

A mixture of 2-ethoxy-6-formylphenylboronic acid (80 g, 0.41 mol), NaOH (16.5 g, 0.41 mol) and cetyltrimethylammonium bromide (CTAB) (7.7 g, 20 mmol) in H_2O (100 mL) and THF (500 mL) was stirred for 0.5 h. After dropwise addition of nitromethane (14 mL, 2,4 mol), the reaction mixture was stirred for additional 3 h, then the mixture was acidified with 2 N HCl to pH 2 and extracted with EtOAc (3 × 300 mL). The combined organic layers were washed with brine (250 mL), dried over anhydrous Na_2SO_4 and concentrated *in vacuo*. The residue was purified by silica gel chromatography eluted with hexane/ethyl acetate to give 7-ethoxy-3-(nitromethyl)benzo[c][1,2]oxaborol-1(3H)-ol as a white solid (92 g, yield 94%). ^{1}H NMR (400 MHz, DMSO-d_6) δ 9.06 (s, 1H), 7.43-7.46 (t, 1H), 7.05-7.07 (d, 1H), 6.87-6.89 (d, 1H), 5.69-5.71 (m, 1H), 5.27-5.31 (m, 1H), 4.51-4.57 (m, 1H), 4.07-4.12 (q, 2H), 1.30-1.34 (t, 3H).

A mixture of 7-ethoxy-3-(nitromethyl)benzo[c][1,2]oxaborol-1(3H)-ol (2 g, 8.43 mmol), Raney Ni (200 mg) and 2 M NH_3/EtOH (10 mL) in ethanol(35 mL) was shaken under an atmosphere of H_2 for 2 h. The mixture was filtered through a bed of Celite and the filtrate was concentrated *in vacuo*. The crude amine was dissolved in EtOAc (10 mL) and 1 N HCl/Et_2O (30 mL) was added. After 1 h, the suspension was filtered and the resulting solid was washed with acetonitrile/hexane (2:1, 2 × 20 mL) to give 3-(aminomethyl)-7-ethoxybenzo[c][1,2]oxaborol-1(3H)-ol hydrochloride **(compound 1)** as a white solid (1.0 g,

yield 49%). ^{1}H NMR (400 MHz, DMSO-d_6) δ 8.89 (s, 1H), 8.22 (bs, 3H), 7.44-7.48 (t, 1H), 7.04-7.06 (d, 1H), 6.88-6.90 (d, 1H), 5.31-5.33 (m, 1H), 4.08-4.13 (q, 2H), 3.39-3.44 (m, 1H), 2.78-2.80 (m, 1H), 1.33-1.36 (t, 3H); ESI-MS *m/z* = 208 $[M + H]^+$; HPLC purity: 98.8% (220 nm).

3-(Aminomethyl)-4-chloro-7-ethoxybenzo[c][1,2]oxaborol-1(3H)-ol hydrochloride (compound 4). To a solution of 7-ethoxy-3-(nitromethyl)benzo[c][1,2]oxaborol-1(3H)-ol (42 g, 0.18 mol) in DMF (200 mL) at 80 °C was added N-chlorosuccinimide (NCS) (24.0 g, 0.18 mol) in DMF (50 mL) over 30 min. The reaction was quenched with an aqueous solution of LiCl (500 mL) and the mixture was extracted by EtOAc (3 × 250 mL). The combined organic layers were dried over anhydrous Na_2SO_4 and concentrated *in vacuo*. The residue was purified by silica gel chromatography eluted with hexane/ethyl acetate to give a mixture of regioisomers as a white solid (39.7 g, containing 18% C6-Cl regioisomer). The mixture was recrystallized from diethyl ether/petroleum ether (1/5) to give 4-chloro-7-ethoxy-3-(nitromethyl)benzo[c][1,2]oxaborol-1(3H)-ol (28 g, yield 57%). ^{1}H NMR (400 MHz, DMSO-d_6) δ 9.32 (s, 1H), 7.40-7.58 (d, 1H), 6.96-6.98 (d, 1H), 5.74-5.77 (d, 1H), 5.31-5.35 (d, 1H), 4.67-4.73 (m, 1H), 4.07-4.12 (q, 2H), 1.28-1.34 (t, 3H). A mixture of 4-chloro-7-ethoxy-3-(nitromethyl)benzo[c][1,2]oxaborol-1(3H)-ol (47 g, 0.17 mol), Raney Ni (2 g) and 2 M NH_3/EtOH (40 mL) in EtOH (200 mL) was stirred under an atmosphere of H_2 for 2 h. The mixture was filtered through a bed of Celite. The filtrate was acidified by using 4.5 N HCl (100 mL). After stirring for 30 min, the mixture was concentrated and the residue was washed with CH_3CN (2 × 50 mL) to give 3-(aminomethyl)-4-chloro-7-ethoxybenzo[c][1,2]oxaborol-1(3H)-ol hydrochloride **(compound 4)** as a white solid (43 g, yield 89%). ^{1}H NMR (400 MHz, DMSO-d_6) δ 9.13 (s, 1 H), 8.18 (bs, 3H), 7.50-7.51 (d, 1H), 6.97-7.00 (d, 1H), 5.36-5.39 (m, 1H), 4.08-4.14 (q, 2H), 3.55-3.59 (m, 1H), 2.90-2.95 (m, 1H), 1.33-1.36 (t, 3H); ESI-MS *m/z* = 242 $[M + H]^+$; HPLC purity: 95.5% (220 nm).

3-(Aminomethyl)-4-bromo-7-ethoxybenzo[c][1,2]oxaborol-1(3H)-ol TFA salt (compound 11). To a mixture of 3-(aminomethyl)-7-ethoxybenzo[c][1,2]oxaborol-1(3H)-ol hydrochloride (300 mg, 1.23 mmol) and triethylamine (622 mg, 6.16 mmol) in DCM (35 mL) at 0 °C was added di-*tert*-butyl dicarbonate (403 mg, 1.85 mmol). The reaction was stirred for 2 h and then quenched with saturated solution of $NaHCO_3$ (45 mL), which was then extracted with EtOAc (3 × 30 mL). The combined organic layers were dried over anhydrous Na_2SO_4 and concentrated *in vacuo* to give *tert*-butyl ((7-ethoxy-1-hydroxy-1,3-dihydrobenzo[c][1,2]oxaborol-3-yl)methyl)carbamate (320 mg, yield 85%). To a solution of

this intermediate (250 mg, 0.81 mmol) and N-bromosuccinimide (173.9 mg, 0.98 mmol) in CH_3CN (50 mL) was added 10 mg of 2,2'-azobis(2-methylpropionitrile). The mixture was stirred at 90 °C for 1 h and then concentrated *in vacuo*, and the resulting residue was purified by prep-HPLC to give *tert*-butyl (4-bromo-7-ethoxy-1-hydroxy-1,3-dihydrobenzo[c][1,2]oxaborol-3-yl)methylcarbamate (200 mg, yield 64%). ^1H NMR (400 MHz, DMSO-d_6) δ 8.90 (s, 1H), 7.55-7.53 (d, 1H), 6.85-6.82 (d, 1H), 5.08-5.07 (d, 1H), 4.11-4.07 (m, 2H), 3.82-3.79 (d, 1H), 3.06-3.03 (m, 1H), 1.39 (s, 9H), 1.30 (t, 3H); ESI-MS *m/z* = 387 $[M + H]^+$. A mixture of *tert*-butyl (4-bromo-7-ethoxy-1-hydroxy-1,3-dihydrobenzo[c][1,2]oxaborol-3-yl)methylcarbamate (200 mg, 51.8 mmol) in trifluoroacetic acid/DCM (1:1, 20 mL) was stirred for 1 h, and then concentrated *in vacuo*. The resulting residue was washed with acetonitrile (2 × 5 mL) to give 3-(aminomethyl)-4-bromo-7-ethoxybenzo[c][1,2]oxaborol-1(3H)-ol TFA salt as a white solid (190 mg, yield 92%). ^1H NMR (400 MHz, DMSO-d_6) δ 9.12 (s, 1H), 8.04 (bs, 3H), 7.62-7.65 (d, 1H), 6.92-6.94 (d, 1H), 5.25-5.27 (m, 1H), 4.08-4.13 (q, 2H), 3.61-3.64 (m, 1H), 2.92-2.99 (m, 1H), 1.33-1.36 (t, 3H); ESI-MS *m/z* = 286 $[M + H]^+$; HPLC purity: 96.0% (220 nm).

Scheme 2

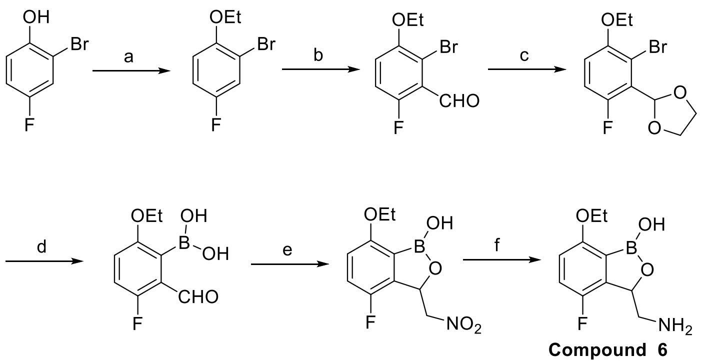

Reagents and conditions: (a) EtBr, *t*-BuOK, DMSO; (b) LDA, DMF, THF, -78 ºC; (c) $HOCH_2CH_2OH$, TsOH, toluene, reflux; (d) (1) *n*-BuLi, (*i*PrO)$_3$B, THF, -78 ºC; (2) 2 N HCl, rt; (e) CH_3NO_2, NaOH, CTAB, water/THF; (f) Raney Ni, H_2, MeOH, then HCl/Et_2O.

3-(Aminomethyl)-7-ethoxy-4-fluorobenzo[c][1,2]oxaborol-1(3H)-ol hydrochloride (Compound 6). To a solution of 2-bromo-4-fluorophenol (40 g, 0.21 mol) and bromoethane (27.4 g, 0.25 mol) in anhydous DMSO (200 mL) was added *t*-BuOK (28.1 g, 0.25 mol) under N_2. The reaction was stirred at room temperature for 3 h. The mixture was diluted with water, acidified to pH 6 with 2 N HCl and extracted with EtOAc (3 × 200 mL). The combined organic layers were washed with brine, dried over Na_2SO_4, the solvent was removed *in vacuo* to give 2-bromo-1-ethoxy-4-fluorobenzene (40 g, yield 87%) as a yellow oil. ^1H NMR (400 MHz, $CDCl_3$) δ 7.25-7.29 (m, 1H), 6.91-6.96 (m, 1H), 6.77-6.81 (m, 1H), 4.01-4.04 (q, 2H),

1.41-1.45 (t, 3H). To a solution of diisopropylamine (11.1 g, 0.11 mol) in dry THF at -78 ^{o}C was added *n*-BuLi (2.5 M in THF, 43.8 mL, 0.11 mol) under N_2. After the mixture was stirred at -78 ^{o}C for 1 h, 2-bromo-1-ethoxy-4-fluorobenzene (20 g, 0.09 mol) was added. The reaction was stirred for 1 h, and DMF (20 g, 0.27 mol) was added. The mixture was further stirred for 30 min, quenched with brine and acidified to pH 5 with 2 N HCl and the two layers were separated. The organic layer was dried over Na_2SO_4 and concentrated *in vacuo* to give 2-bromo-3-ethoxy-6-fluorobenzaldehyde (18 g, yield 80%). ^{1}H NMR (400 MHz, $CDCl_3$) δ 10.33 (s, 1H), 7.04-7.06 (m, 2H), 4.04-4.10 (q, 2H), 1.41-1.45 (t, 3H). To a solution of 2-bromo-3-ethoxy-6-fluorobenzaldehyde (22 g, 0.089 mol), glycol (8.29 g, 0.134 mol) in toluene (300 mL) was added *p*-toluenesulfonic acid (1.69 g, 0.0089 mol). The reaction flask had a Dean-Stark condenser attached and the reaction mixture was refluxed to remove water for 4 h. The reaction mixture was cooled to room temperature and washed with aqueous $NaHCO_{3.}$ The organic layer was dried over Na_2SO_4 and concentrated *in vacuo* to give 2-(2-bromo-3-ethoxy-6-fluorophenyl)-1,3-dioxolane (18 g, yield 69%). ^{1}H NMR (400 MHz, $CDCl_3$) δ 6.95-7.00 (m, 1H), 6.83-6.86 (m, 1H), 6.40(s, 1H), 4.21-4.25 (m, 4H), 4.01-4.06 (q, 2H), 1.41-1.45 (t, 3H). To a solution of 2-(2-bromo-3-ethoxy-6-fluorophenyl)-1,3-dioxolane (3.0 g, 10.3 mmol) in anhydrous THF (250 mL) at -78 ^{o}C was added *n*-BuLi (2.5 M in THF, 5.4 mL, 13.5 mol) dropwise under N_2. After the mixture was stirred at -78 ^{o}C for 2 h, triisopropyl borate (3.85 g, 20.5 mmol) was added dropwise. The resulting mixture was stirred at -78 ^{o}C for 4 h. The reaction was quenched by adding saturated NH_4Cl solution (150 mL), and the mixture was extracted with EtOAc (3 × 150 mL). The combined organic layers were washed with water (100 mL) and brine (100 mL), dried over anhydrous Na_2SO_4 and concentrated to give diisopropyl (2-(1,3-dioxolan-2-yl)-6-ethoxy-3-fluorophenyl)boronate (2.5 g, yield 71%), which was used in the next step without further purification. To a solution of this crude boronate (2.5 g, 7.35 mmol) in THF (50 mL) a 100 mL of 2 N HCl was added slowly. The reaction mixture was stirred for 3h then it was basified with the addition of 20% NaOH to pH 12 and then washed with EtOAc (2 × 100 mL). The aqueous layer was acidified by using 2 N HCl to pH 2 and then extracted with EtOAc (3 × 100 mL). The combined organic layers were dried over anhydrous Na_2SO_4 and concentrated *in vacuo* to give (6-ethoxy-3-fluoro-2-formylphenyl)boronic acid (0.8 g, yield 51%), which was used in the next step without further purification. A mixture of (6-ethoxy-3-fluoro-2-formylphenyl)boronic acid (0.8 g, 3.77 mmol), NaOH (0.15 g, 3.77 mmol) and CTAB (0.07 g, 0.19 mmol) in H_2O (50 mL) and THF (100 mL) was stirred for 30 min then nitromethane (1.38 g, 22.6 mmol)

was added dropwise and the reaction mixture was stirred for 3 h. The mixture was subsequently acidified with 2 N HCl to pH 2 and extracted with EtOAc (3 × 100 mL). The combined organic layers were washed with brine (100 mL), dried over anhydrous Na_2SO_4 and concentrated *in vacuo* to give 7-ethoxy-4-fluoro-3-(nitromethyl)benzo[c][1,2]oxaborol-1(3H)-ol as a yellow solid (0.5 g, yield 52%). ^{1}H NMR (400 MHz, DMSO-d_6) δ 9.32 (s, 1H), 7.22-7.27 (m, 1H), 6.88-6.91 (m, 1H), 5.82-5.86 (m, 1H), 5.14-5.18 (m, 1H), 4.63-4.69 (m, 1H), 4.03-4.08 (q, 2H), 1.27-1.31 (t, 3H). A mixture of 7-ethoxy-4-fluoro-3-(nitromethyl)benzo[c][1,2]oxaborol-1(3H)-ol (4.0 g, 15.6 mmol) and Raney Ni (800 mg) in MeOH (150 mL) was stirred under 50 psi of H_2 for 2 h. The mixture was filtered through a bed of Celite and the filtrate was concentrated *in vacuo*. The crude amine was re-dissolved in EtOAc (10 mL) and 1 M HCl/Et_2O (60 mL) was added. After 1 h, the suspension was filtered and the resulting solid was washed with acetonitrile to give 3-(aminomethyl)-7-ethoxy-4-fluorobenzo[c][1,2]oxaborol-1(3H)-ol hydrochloride (**compound 6**) as a white solid (2.5 g, yield 61%). ^{1}H NMR (400 MHz, DMSO-d_6) δ 9.15 (s, 1H), 8.35 (bs, 3H), 7.23-7.28 (m, 1H), 6.89-6.93 (m, 1H), 5.47-5.50 (m, 1H), 3.34-3.38 (m, 1H), 2.86-2.89 (m, 1H), 4.04-4.09 (q, 2H), 1.29-1.33 (t, 3H); ESI-MS m/z = 226 $[M + H]^+$; HPLC purity: 96.7% (220 nm).
Scheme 3

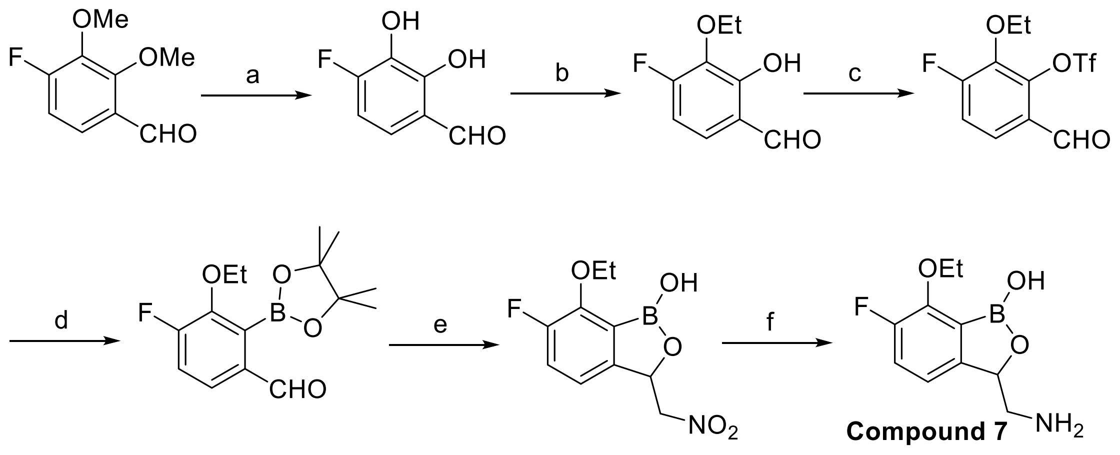

Reagents and conditions: (a) BBr_3, DCM, -78 ºC; (b) EtBr, $NaOBu^t$, DMSO; (c) Tf_2O, pyridine, DCM, 0 ºC; (d) bis(pinacolato)diboron, $PdCl_2$(dppf), KOAc, THF, reflux; (e) CH_3NO_2, NaOH, THF; (f) $Pd(OH)_2$, H_2, NH_3/MeOH, then HCl/MeOH.

3-(Aminomethyl)-7-ethoxy-6-fluorobenzo[c][1,2]oxaborol-1(3H)-ol hydrochloride (compound 7). To a solution of 4-fluoro-2,3-dimethoxybenzaldehyde (7.0 g, 38.0 mmol) in dry DCM (150 mL) at -78 ºC was added dropwise BBr_3 (23.8 g, 95.0 mmol) in DCM (30 mL). The reaction mixture was allowed to reach room temperature and stirred for 18 h. Then

the mixture was cooled to -78 °C, quenched with a mixture of methanol (10 mL) and water (50 mL), and stirred at room temperature for 30 min. Precipitated solid was separated by filtration and washed with cold DCM. The organic layer was concentrated to yield 4-fluoro-2,3-dihydroxybenzaldehyde (5.2 g, yield 88%). ^{1}H NMR (400 MHz, $CDCl_3$) δ 11.38 (s, 1H), 9.84 (s, 1H), 7.15 (dd, 1H), 6.81 (m, 1H), 5.47 (s, 1H); ESI-MS *m/z* = 155 $[M - H]^-$.

To a solution of 2,3-dihydroxy-4-fluorobenzaldehyde (3.0 g, 19.2 mmol) in DMSO (100 mL) *t*-BuONa (3.69 g, 38.46 mmol) was added and after being stirred for 15 min, iodoethane was added dropwise. The reaction was stirred for 18 h and then the mixture was poured onto crushed ice (200 mL) and acidified with 2.5 N HCl to pH 3. After extraction with ethyl acetate (2 × 100 mL), the combined organic layers were dried over anhydrous Na_2SO_4 and concentrated *in vacuo*. The residue was purified by silica gel chromatography eluted with hexane and EtOAc (95:5) to give 3-ethoxy-4-fluoro-2-hydroxybenzaldehyde as a crystalline solid (2.3 g, 65%). ^{1}H NMR (400 MHz, $CDCl_3$) δ 11.36 (s, 1H), 9.83 (s, 1H), 7.15-7.39 (m, 1H), 6.77 (m, 1H), 4.22 (q, 2H), 1.40 (t, 3H); ESI-MS *m/z* = 183 $[M - H]^-$.

To a mixture of 3-ethoxy-4-fluoro-2-hydroxybenzaldehyde (2.21 g, 12.0 mmol) and pyridine (1.99 g, 24.0 mmol) in DCM (30.0 mL) at 0 °C trifluoromethanesulfonic anhydride (4.06, 14.4 mmol) in DCM (5.0 mL) was added dropwise. The reaction was stirred at 0 °C for 2 h and then at room temperature for a further 3 h. The mixture was diluted with DCM (40 mL), washed with 2 N HCl, brine and dried over anhydrous Na_2SO_4, and the solvent was removed under reduced pressure to give 2-ethoxy-3-fluoro-6-formylphenyl trifluoromethanesulfonate as a light yellow liquid (3.3 g, 87%). ^{1}H NMR (400 MHz, $CDCl_3$) δ 10.15 (s, 1H), 7.66 (dd, 1H), 7.22 – 7.28 (m, 1H), 4.36 (q, 2H), 1.47 (t, 3H). To a solution of 2-ethoxy-3-fluoro-6-formylphenyl trifluoromethanesulfonate (2.2 g, 6.96 mmol) in dry THF (35 mL) were added bis(pinacolato)diboron (2.13 g, 8.4 mmol), $PdCl_2$(dppf) (367 mg, 0.5 mmol) and potassium acetate (1.37 g, 14.0 mmol). After purged with N_2 for 15 min, the reaction mixture was heated under reflux for 24 h. The mixture was cooled to room temperature, diluted with ethyl acetate (40 mL) and filtered through Celite. The solvent was removed under reduced pressure. The residue was purified by silica gel chromatography eluted with hexane and EtOAc (9:1) to give 3-ethoxy-4-fluoro-2-(4,4,5,5-tetramethyl-1,3,2-dioxaborolan-2-yl)benzaldehyde as an off-white solid (850 mg, 42%). ^{1}H NMR (400 MHz, $CDCl_3$) δ 9.87 (s, 1H), 7.51 (dd, 1H), 7.22 (dd, 1H), 4.20 (q, 2H), 1.46 (s, 12H), 1.40 (t, 3H); ESI-MS *m/z* = 295 $[M + H]^+$. To a cooled solution of sodium hydroxide (80 mg, 2.0 mmol) in water (3 mL) at 0 °C was added nitromethane (244 mg, 4.0 mmol) and after stirring for 10 min 3-ethoxy-4-

fluoro-2-(4,4,5,5-tetramethyl-1,3,2-dioxaborolan-2-yl)benzaldehyde (588 mg, 2.0 mmol) in THF (5 mL) was added. The reaction was stirred for a further 1 h at 0 °C and then 2 h at room temperature. The mixture was acidified with 2.5 N HCl (1 mL) and extracted with ethyl acetate (2 × 20 mL) and the combined organic extracts were washed with brine and dried over anhydrous Na_2SO_4 with the solvent being removed under reduced pressure. The resulting residue was purified by silica gel chromatography eluted with DCM and MeOH (95:5) to give 7-ethoxy-6-fluoro-3-(nitromethyl)benzo[c][1,2]oxaborol-1(3H)-ol as a solid (350 mg, 69%). ^{1}H NMR (400 MHz, DMSO-d_6) δ 7.40 (dd, 1H), 7.16 (dd, 1H), 5.74 (d, 1H), 5.30 (dd, 1H), 4.62 (dd, 1H), 4.35 (q, 2H), 1.28 (t, 3H); ESI-MS *m/z* = 254 $[M - H]^-$.
To a solution of 7-ethoxy-6-fluoro-3-(nitromethyl)benzo[c][1,2]oxaborol-1(3H)-ol (320 mg, 1.25 mmol) in methanol (5 mL) were added 5 mL of 2 M NH_3/MeOH and 160 mg of $Pd(OH)_2$/C. The reaction was hydrogenated at 45 psi of H_2 for 18 h and catalyst was removed by filtration through Celite and the filtrate was concentrated to generate an off-white solid (250 mg). This solid was dissolved in methanol (3 mL) and 3.0 mL of 1.2 M HCl in methanol was added. After stirring for 3 h, excess HCl and solvent were removed under reduced pressure and the product was titrated with ether to give 3-(aminomethyl)-7-ethoxy-6-fluorobenzo[c][1,2]oxaborol-1(3H)-ol hydrochloride **(compound 7)** as a white solid (140 mg, 43%). ^{1}H NMR (400 MHz, DMSO-d_6) δ 9.43 (s, 1H), 8.13 (br, 3H), 7.40 (dd, 1H), 7.16 (dd, 1H), 5.32 (m, 1H), 4.35 (q, 2H), 3.43 (m, 1H), 2.92 (m, 1H), 1.29 (t, 3H); ESI-MS *m/z* = 226 $[M + H]^+$; HPLC purity: 95.8% (220 nm).
Scheme 4

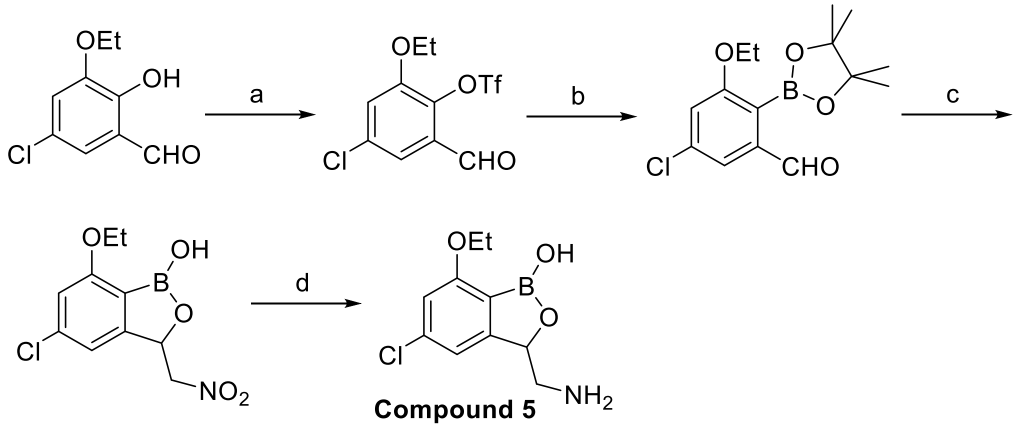

Reagents and conditions: (a) Tf_2O, pyridine, DCM, 0 ºC; (b) bis(pinacolato)diboron, $PdCl_2$(dppf), KOAc, dioxane, 100 ºC; (c) CH_3NO_2, NaOH, CTAB, water/THF; (d) Raney Ni, H_2, NH_3/EtOH, then HCl/Et_2O.

3-(Aminomethyl)-5-chloro-7-ethoxybenzo[c][1,2]oxaborol-1(3H)-ol hydrochloride (compound 5). To a solution of 5-chloro-3-ethoxy-2-hydroxybenzaldehyde (2.0 g, 10.0 mmol) in pyridine (2 mL) and DCM (20 mL) at 0 °C was added trifluoromethanesulfonic anhydride (1 mL) dropwise. The reaction was stirred at 0 °C for 1 h and then quenched with ice-water. The organic layer was washed with a saturated solution of $NaHCO_3$ (20 mL) and brine (20 mL), dried over anhydrous Na_2SO_4 and concentrated *in vacuo*. The residue was purified by silica gel chromatography eluted with petroleum ether/ethyl acetate (10:1) to give 4-chloro-2-ethoxy-6-formylphenyl trifluoromethanesulfonate (2.0 g, yield 60%). A mixture of 4-chloro-2-ethoxy-6-formylphenyl trifluoromethanesulfonate (330 mg, 1.0 mmol), KOAc (350 mg, 2.0 mmol), bis(pinacolato)diborane (600 mg, 2.0 mmol) and $PdCl_2$(dppf) (65 mg, 0.08 mmol) in dioxane (30 mL) was degassed for 15 min with N_2 and stirred at 100 °C for 3 h. After the reaction was quenched with ice-water, the mixture was then extracted with EtOAc (3 × 30 mL). The combined organic layers were washed with a saturated solution of $NaHCO_3$ (20 mL) and brine (20 mL), dried over anhydrous Na_2SO_4 and concentrated *in vacuo*. The residue was purified by silica gel chromatography eluted with petroleum ether/ethyl acetate (10:1) to give 5-chloro-3-ethoxy-2-(4,4,5,5-tetramethyl-1,3,2-dioxaborolan-2-yl)benzaldehyde (150 mg, yield 48%). A mixture of 5-chloro-3-ethoxy-2-(4,4,5,5-tetramethyl-1,3,2-dioxaborolan-2-yl)benzaldehyde (310 mg, 1.0 mmol), NaOH (40 mg, 1 mmol) and CTAB (5.0 mg, 0.05 mmol) in H_2O (2 mL) and THF (10 mL) was stirred at room temperature for 30 min. After dropwise addition of nitromethane (0.2 mL, 2.0 mmol), the reaction was stirred for 3 h. The mixture was then acidified with 2 N HCl to pH 2 and extracted with EtOAc (3 × 30 mL). The combined organic layers were washed with brine (25 mL), dried over anhydrous Na_2SO_4 and concentrated *in vacuo* to give 5-chloro-7-ethoxy-3-(nitromethyl)benzo[c][1,2]oxaborol-1(3H)-ol (100 mg, yield 37%). ^{1}H NMR (400 MHz, DMSO-d_6) δ 9.20 (s, 1 H), 7.22 (s, 1H), 6.96 (s, 1H), 5.69-5.72 (m, 1H), 5.30-5.34 (m,1H), 4.61-4.67 (m, 1H), 4.10-4.15 (q, 2H), 1.31-1.34 (t, 3H). A mixture of 5-chloro-7-ethoxy-3-(nitromethyl)benzo[c][1,2]oxaborol-1(3H)-ol (270 mg, 1.0 mmol), Raney Ni (125 mg) and 2 M NH_3/EtOH (2 mL) in EtOH (10 mL) was shaken under an atmosphere of H_2 for 2 h. The mixture was filtered through a bed of Celite and the filtrate was concentrated in vacuo. The crude amine was dissolved in EtOAc (2 mL) and 1 M HCl/Et_2O (20 mL) was added. After 1 h, the suspension was filtered and the solid was washed with hexane to give 3-(aminomethyl)-5-chloro-7-ethoxybenzo[c][1,2]oxaborol-1(3H)-ol hydrochloride (**compound 5**) as a yellow solid (100 mg, yield 36%). ^{1}H NMR (400 MHz, DMSO-d_6) δ 9.06 (s, 1H),

8.18 (bs, 3H), 7.22 (s, 1H), 6.96 (s, 1H), 5.27-5.29 (m, 1H), 4.10-4.13 (q, 2H), 3.40-3.47 (m, 1H), 2.87-2.92 (m, 1H), 1.30-1.36 (t, 3H); ESI-MS *m/z* = 242 $[M + H]^+$; HPLC purity: 97.6% (220 nm).

Scheme 5

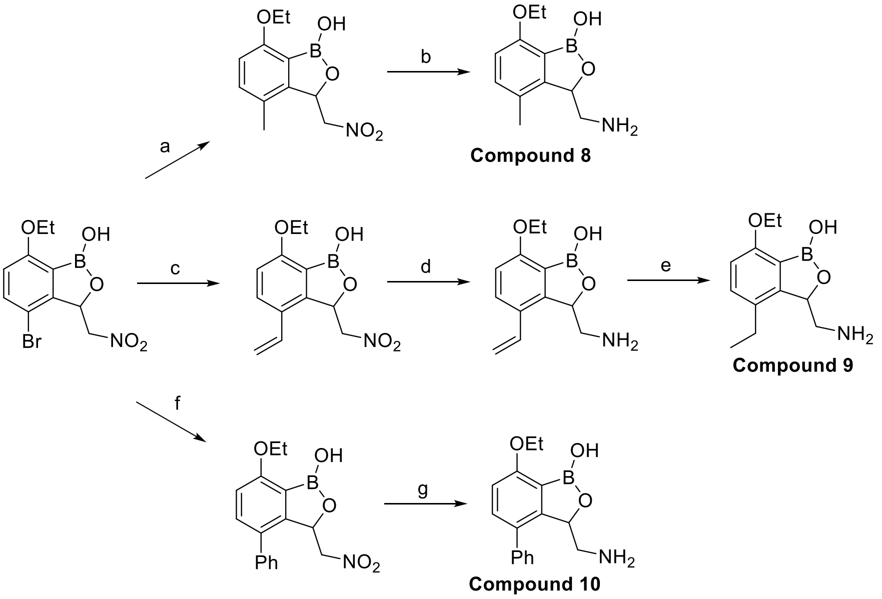

Reagents and conditions: (a) $SnMe_4$, $Pd(PPh_3)_4$, DMF, 90 ºC; (b) Raney Ni, H_2, NH_3/EtOH, then HCl/EtOH; (c) (*n*-Bu)$_3$SnCH=CH$_2$, $Pd(PPh_3)_4$, DMF, Microwave, 100 ºC; (d) Raney Ni, H_2, NH_3/EtOH, then HCl/Et_2O; (e) Pd/C, H_2, EtOH, then HCl/Et_2O; (f) (*n*-Bu)$_3$SnPh, $Pd(PPh_3)_4$, DMF, Microwave, 100 ºC; (g) Raney Ni, H_2, NH_3/EtOH, then HCl/Et_2O.

3-(Aminomethyl)-7-ethoxy-4-methylbenzo[c][1,2]oxaborol-1(3H)-ol hydrochloride (compound 8). A mixture of 4-bromo-7-ethoxy-3-(nitromethyl)benzo[c][1,2]oxaborol-1(3H)-ol (200 mg, 0.63 mmol), tetramethylstannane (341 mg, 1.91 mmol) and $Pd(PPh_3)_4$ (20 mg) in DMF (35 mL) was stirred overnight at 90 ^oC under N_2. After the reaction was quenched by adding ice-water, the mixture was extracted with ethyl acetate (3 × 30 mL). The combined extracts were dried over anhydrous Na_2SO_4, and concentrated *in vacuo*. The residue was purified by prep-TLC eluted with DCM and MeOH (50:1) to give 7-ethoxy-4-methyl-3-(nitromethyl)benzo[c][1,2]oxaborol-1(3H)-ol (72 mg, yield 45%). ^{1}H NMR (400 MHz, DMSO-*d$_6$*) δ 9.00 (s, 1H), 7.21-7.23 (d, 1H), 6.81-6.83 (d, 1H), 5.75-5.77 (m, 1H), 5.24-5.27 (m, 1H), 4.44-4.50 (m, 1H), 4.03-4.08 (q, 2H), 2.25 (s, 3H), 1.33-1.29 (t, 3H).

A mixture of 7-ethoxy-4-methyl-3-(nitromethyl)benzo[c][1,2]oxaborol-1(3H)-ol (80 mg, 0.32 mmol), Raney Ni (50 mg) and 2 M NH_3/EtOH (2 mL) in EtOH (10 mL) was stirred under an atmosphere of H_2 for 2 h. After the mixture was filtrated, the filtrate was acidified by using 4.5 N HCl (15 mL) and stirred for 30 min. The resulting mixture was then concentrated *in*

vacuo and the residue was washed with CH_3CN (2 × 3 mL) to give 3-(aminomethyl)-7-ethoxy-4-methylbenzo[c][1,2]oxaborol-1(3H)-ol hydrochloride (**compound 8**) as a white solid (39 mg, yield 48%). ^{1}H NMR (400 MHz, DMSO-d_6) δ 8.80 (s, 1H), 8.15 (bs, 3H), 7.22-7.24 (d, 1H), 6.81-6.83 (d, 1H), 5.35-5.37 (m, 1H), 4.03-4.08 (q, 2H), 3.28-3.36 (m, 1H), 2.70-2.73 (m, 1H), 2.23 (s, 3H), 1.30-1.34 (t, 3H); ESI-MS *m/z* = 222 $[M + H]^+$; HPLC purity: 98.8% (220 nm).

3-(Aminomethyl)-7-ethoxy-4-ethylbenzo[c][1,2]oxaborol-1(3H)-ol hydrochloride (compound 9). A mixture of 4-bromo-7-ethoxy-3-(nitromethyl)benzo[c][1,2]oxaborol-1(3H)-ol (900 mg, 2.85 mmol), vinyltributyltin (5.2 g, 16.4 mmol) and $Pd(PPh_3)_4$ (230 mg, 0.2 mmol) in DMF (45 mL) was degassed with N_2 for 15 min. The reaction mixture was stirred at 100 ^oC in a microwave reactor (Biotage) for 30 min. After the reaction was quenched with ice-water, the mixture was extracted with EtOAc (3 × 30 mL). The combined organic layers were washed with water (20 mL) and brine (20 mL), dried over anhydrous Na_2SO_4, and concentrated *in vacuo*. The residue was purified by silica gel chromatography eluted with DCM to give 7-ethoxy-3-(nitromethyl)-4-vinylbenzo[c][1,2]oxaborol-1(3H)-ol (650 mg, yield 87%). ^{1}H NMR (400 MHz, DMSO-d_6) δ 9.10 (s, 1 H), 7.64-7.66 (d, 1H), 6.93-6.95 (d, 1H), 6.77-6.84 (m, 1H), 5.93-5.96 (d, 1H), 5.69-5.73 (d, 1H), 5.28-5.31 (d, 1H), 5.10-5.14 (d, 1H), 4.44-4.49 (m, 1H), 4.09-4.14 (q, 2H), 1.32-1.35 (t, 3H). A mixture of 7-ethoxy-3-(nitromethyl)-4-vinylbenzo[c][1,2]oxaborol-1(3H)-ol (205 mg, 0.78 mmol), Raney Ni (50 mg) and 2 M NH_3/EtOH (5 mL) in EtOH (10 mL) was shaken under an atmosphere of H_2 for 2 h. The mixture was filtered through Celite and the filtrate was concentrated *in vacuo*. The crude amine was then dissolved in 2 mL of EtOAc and then 20 mL of 1 M HCl/Et_2O was added. After 1 h, the suspension was filtered to give 3-(aminomethyl)-7-ethoxy-4-vinylbenzo[c][1,2]oxaborol-1(3H)-ol, which was used directly in the next step without further purification. A suspension of 3-(aminomethyl)-7-ethoxy-4-vinylbenzo[c][1,2]oxaborol-1(3H)-ol (175 mg, 0.75 mmol) and Pd/C (40 mg) in EtOH (5 ml) was shaken under an atmosphere of H_2 for 2 h. The mixture was filtered through a bed of Celite and the filtrate was concentrated *in vacuo*. The residue was then purified by prep-HPLC followed by treatment with conc. HCl to give 3-(aminomethyl)-7-ethoxy-4-ethylbenzo[c][1,2]oxaborol-1(3H)-ol hydrochloride (**compound 9**) as a white solid (23 mg, yield 11%). ^{1}H NMR (400 MHz, DMSO-d_6) δ 8.81 (s, 1 H), 8.18 (bs, 3H), 7.29-7.31 (d, 1H), 6.68-6.88 (d, 1H), 5.38-5.40 (d, 1H), 4.04-4.09 (q, 2H), 3.30-3.35 (m, 1H), 2.66-2.71 (m, 1H), 1.31-1.34 (t, 3H), 1.15-1.17 (t, 3H); ESI-MS *m/z* = 236 $[M + H]^+$; HPLC purity: 99.7% (220 nm).

3-(Aminomethyl)-7-ethoxy-4-phenylbenzo[c][1,2]oxaborol-1(3H)-ol hydrochloride (compound 10). A mixture of 4-bromo-7-ethoxy-3-(nitromethyl)benzo[c][1,2]oxaborol-1(3H)-ol (315 mg, 1.0 mmol), tributylphenylstannane (750 mg, 2.0 mmol) and $Pd(Ph_3P)_4$ (cat.) in DMF (15 mL) was degassed for 15 min with N_2 and then stirred at 100 °C for 30 min in a microwave reactor (Biotage). After the reaction was quenched with ice-water, the resulting mixture was extracted with EtOAc (3 × 40 mL). The combined organic layers were washed with water (20 mL) and brine (20 mL), dried over anhydrous Na_2SO_4 and then concentrated *in vacuo*. The residue was purified by prep-HPLC followed by treating with concentrated HCl to give 7-ethoxy-3-(nitromethyl)-4-phenylbenzo[c][1,2]oxaborol-1(3H)-ol as a white solid (60 mg, yield 20%). ^{1}H NMR (400 MHz, DMSO-d_6) δ 9.13 (s, 1 H), 7.46-7.49 (m, 5H), 7.34-7.48 (m, 2H), 6.17-6.20 (m, 1H), 4.88-4.92 (m, 1H), 4.21-4.25 (m, 1H), 4.05-4.16 (q, 2H), 1.34-1.37 (t, 3H). A mixture of 7-ethoxy-3-(nitromethyl)-4-phenylbenzo[c][1,2]oxaborol-1(3H)-ol (60 mg, 0.19 mmol), Raney Ni (~25 mg) and 2 M NH_3/EtOH (2 mL) in EtOH (10 mL) was shaken under an atmosphere of H_2 for 2 h. The mixture was filtered through a bed of Celite and the filtrate was concentrated *in vacuo*. The crude amine was dissolved in EtOAc (1 mL) and 1 M HCl/Et_2O (5 mL) was added. After 1 h, the suspension was filtered and the resulting solid was washed with hexane to give 3-(aminomethyl)-7-ethoxy-4-phenylbenzo[c][1,2]oxaborol-1(3H)-ol hydrochloride (**compound 10**) as a yellow solid (30 mg, yield 50%). ^{1}H NMR (400 MHz, DMSO-d_6) δ 8.89 (s, 1 H), 8.04 (bs, 3H), 7.43-7.46 (d, 5H), 7.36-7.38 (d, 1H), 6.98-7.00 (d, 1H), 5.78-5.81 (d, 1H), 4.09-4.14 (q, 2H), 2.56-2.59 (m, 1H), 2.24-2.30 (m, 1H), 1.33-1.36 (t, 3H); ESI-MS *m/z* = 284 $[M + H]^+$; HPLC purity: 98.0% (220 nm).

Scheme 6

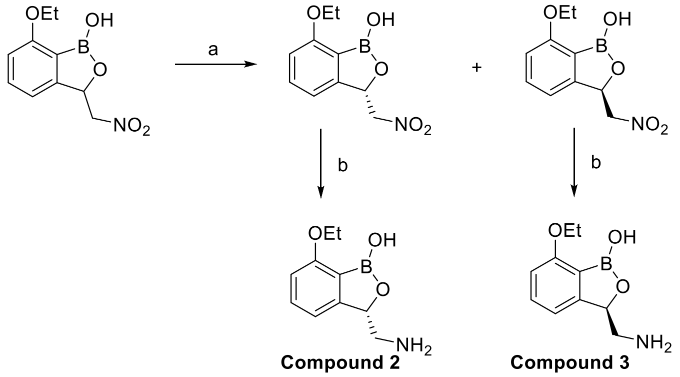

Reagents and conditions: (a) chiral HPLC separation; (b) Raney Ni, H_2, MeOH, then HCl/MeOH.

(S)-3-(Aminomethyl)-7-ethoxybenzo[c][1,2]oxaborol-1(3H)-ol hydrochloride (compound 2). 58 g of racemic 7-ethoxy-3-(nitromethyl)benzo[c][1,2]oxaborol-1(3H)-ol dissolved in methanol (80 mg/mL) was resolved via chiral HPLC using a Chiralpak AD column (20 μM column) eluted with carbon dioxide and methanol mobile phase. Two peaks, (S)-7-ethoxy-3-(nitromethyl)benzo[c][1,2]oxaborol-1(3H)-ol and (R)-7-ethoxy-3-(nitromethyl)benzo[c][1,2]oxaborol-1(3H)-ol were collected. Analysis of the pooled fractions using a Chiralpak AD-H analytical column and the same mobile phase provided the (S) enantiomer [20.5 g, retention time = 1.19 min] with >98% ee, and the (R) enantiomer [19.1 g, retention time = 1.76 min] with >98% ee. (S)-7-ethoxy-3-(nitromethyl)benzo[c][1,2]oxaborol-1(3H)-ol (5.1 g) was hydrogenated with Raney Ni (4 g) in MeOH (200 mL) under an atmosphere of H_2 (50 psi) for 2 h. The mixture was filtered through a bed of Celite, and the filtrate was concentrated *in vacuo*. The crude amine was dissolved in MeOH (30 mL), and then treated with 2 N HCl (3 mL). After 1 h, the solvent was removed *in vacuo* and the solid was washed with *n*-hexane, and was filtered and dried *in vacuo* to give (S)-3-(aminomethyl)-7-ethoxybenzo[c][1,2]oxaborol-1(3H)-ol hydrochloride (**compound 2**) as a white solid (3.0 g). ^{1}H NMR (400 MHz, DMSO-d_6) δ 8.87 (s, 1H), 8.31 (bs, 3H), 7.41-7.45 (t, 1H), 7.01-7.03 (d, 1H), 6.85-6.87 (d, 1H), 5.28-5.31 (m, 1H), 4.05-4.10 (q, 2H), 3.36-3.38 (m, 1H), 2.75-2.77 (m, 1H), 1.29-1.33 (t, 3H); ESI-MS *m/z* = 208 $[M + H]^+$; HPLC purity: 97.4% (220 nm). Analysis of the BOC-derivatized material using a Chiralpak AD-H analytical column showed the (S) enantiomer with a retention time of 4.18 min and >98% ee.

(R)-3-(aminomethyl)-7-ethoxybenzo[c][1,2]oxaborol-1(3H)-ol hydrochloride (compound 3). Compound 3 was obtained (3.2 g, yield 63%) using the same procedure as described above, starting from (R)-7-ethoxy-3-(nitromethyl)benzo[c][1,2]oxaborol-1(3H)-ol. ^{1}H NMR (400 MHz, DMSO-d_6) δ 8.87 (s, 1H), 8.31 (bs, 3H), 7.41-7.45 (t, 1H), 7.01-7.03 (d, 1H), 6.85-6.87 (d, 1H), 5.29-5.31 (m, 1H), 4.05-4.10 (q, 2H), 3.36-3.38 (m, 1H), 2.75 (m, 1H), 1.30-1.33 (t, 3H); ESI-MS *m/z* = 208 $[M + H]^+$; HPLC purity: 97.8% (220 nm). Analysis of the BOC-derivatized material using a Chiralpak AD-H analytical column showed the (S) enantiomer with a retention time of 4.93 min and >98% ee.

Scheme 7

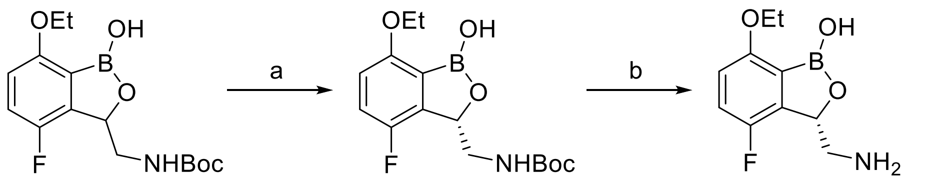

Compound 12

Reagents and conditions: (a) chiral HPLC separation; (b) HCl/MeOH.

(S)-3-(Aminomethyl)-7-ethoxy-4-fluorobenzo[c][1,2]oxaborol-1(3H)-ol hydrochloride (compound 12). 4.5 g of racemic *tert*-butyl ((7-ethoxy-4-fluoro-1-hydroxy-1,3-dihydrobenzo[c][1,2]oxaborol-3-yl)methyl)carbamate dissolved in ethanol (100 mg/mL) was resolved *via* chiral HPLC using a ChiralCel OZ-H column, eluted with carbon dioxide and hexane/ethanol (1:1). 2.2 g of the (S) enantiomer was obtained. Removal of Boc-group followed by lyophilization provided (S)-3-(Aminomethyl)-7-ethoxy-4-fluorobenzo[c][1,2]oxaborol-1(3H)-ol hydrochloride as a white solid (1.4 g). ^{1}H NMR (400 MHz, DMSO-d_6) δ 9.17 (s, 1H), 8.31 (bs, 3H), 7.21-7.28 (t, 1H), 6.89-6.92 (m, 1H), 5.46-5.49 (m, 1H), 4.00-4.09 (q, 2H), 3.29-3.40 (m, 1H), 2.85-2.91 (m, 1H), 1.29-1.32 (t, 3H); ESI-MS *m/z* = 226 $[M + H]^+$; HPLC purity: 98.1% (220 nm). Analysis of the BOC-derivatized material using a ChiralCel OZ-H analytical column showed the (S) enantiomer with a retention time of 3.31 min and 98.1% ee. The (R) enantiomer (2.0 g) had a retention time of 2.66 min and 99.5% ee.

Scheme 8

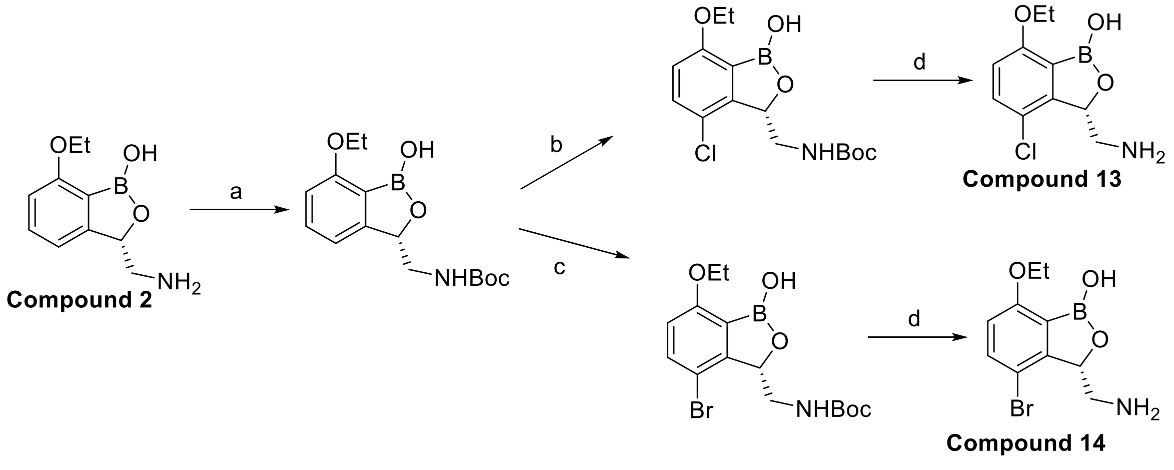

Reagents and conditions: (a) chiral HPLC separation; (b) di-*tert*-butyl dicarbonate, TEA, DCM, 0 ºC; (b) NCS, CH_3CN, 90 ºC; (c) NBS, CH_3CN, 90 ºC; (d) HCl/EtOAc.

(S)-3-(Aminomethyl)-4-chloro-7-ethoxybenzo[c][1,2]oxaborol-1(3H)-ol hydrochloride (compound 13). To a mixture of (S)-3-(aminomethyl)-7-ethoxybenzo[c][1,2]oxaborol-1(3H)-ol hydrochloride (8.0 g, 32.8 mmol) and triethylamine (16.6 g, 164 mmol) in DCM

(300 mL) at 0 °C was added di-*tert*-butyl dicarbonate (10.72 g, 49.2 mmol). The mixture was stirred for 2 h at room temperature. After quenched with saturated solution of $NaHCO_3$, the reaction mixture was extracted with EtOAc. The organic layer was dried over anhydrous Na_2SO_4, and concentrated *in vacuo*. The crude product was washed with *n*-hexane and filtered to give *tert*-butyl (S)-((7-ethoxy-1-hydroxy-1,3-dihydrobenzo[c][1,2]oxaborol-3-yl)methyl)carbamate as a white solid (8 g, yield 79%). ^{1}H NMR (400 MHz, DMSO-d_6) δ 8.70 (s, 1H), 7.35-7.38 (t, 1H), 6.89-6.91 (d, 1H), 6.80-6.82 (d, 1H), 5.03-5.06 (m, 1H), 4.04-4.10 (q, 2H), 3.15-3.16 (d, 1H), 2.98-3.04 (m, 1H), 1.29-1.35 (m, 12H). A solution of *tert*-butyl (S)-((7-ethoxy-1-hydroxy-1,3-dihydrobenzo[c][1,2]oxaborol-3-yl)methyl)carbamate (11.3 g, 36.6 mmol), NCS (4.89 g, 36.6 mmol) and 500 mg 2,2'-azobis(2-methylpropionitrile) in CH_3CN (2000 mL) was stirred at 90 °C for 1 h. The reaction mixture was concentrated *in vacuo* and the residue was purified by prep HPLC eluted with water/acetonitrile gradient (0.025% TFA) to give *tert*-butyl (S)-((4-chloro-7-ethoxy-1-hydroxy-1,3-dihydrobenzo[c][1,2]oxaborol-3-yl)methyl)carbamate as a white solid (7.4 g, yield 58%). ^{1}H NMR (400 MHz, DMSO-d_6) δ 8.90 (s, 1H), 7.39-7.41 (d, 1H), 6.88-6.91 (d, 1H), 6.81 (m, 1H), 5.15-5.16 (d, 1H), 4.06-4.11 (m, 2H), 3.74-3.77 (d, 1H), 3.01-3.08 (m, 1H), 1 .31-1.33 (m, 12H). A mixture of *tert*-butyl (S)-((4-chloro-7-ethoxy-1-hydroxy-1,3-dihydrobenzo[c][1,2]oxaborol-3-yl)methyl)carbamate (7.4 g, 21.7 mmol) in HCl/EtOAc (250 ml) was stirred at room temperature for 2 h, then it was concentrated to dryness and the solid was washed with *n*-hexane. The solid was filtered and dried *in vacuo* to give (S)-3-(aminomethyl)-4-chloro-7-ethoxybenzo[c][1,2]oxaborol-1(3H)-ol hydrochloride **(compound 13)** as a white solid (5.57 g, yield 93%). ^{1}H NMR (400 MHz, DMSO-d_6) δ 9.16 (s, 1H), 8.30 (bs, 3H), 7.48-7.51 (d, 1H), 6.97-6.99 (d, 1H), 5.38-5.40 (m, 1H), 4.08-4.13 (q, 2H), 3.56-3.58 (d, 2H), 2.90 (m, 2H), 1.32-1.36 (t, 3H). ESI-MS *m/z* = 242 $[M + H]^+$; HPLC purity: 99.6% (220 nm). Analysis of its BOC-derivatized material using a Chiralpak AD-H analytical column showed the (S) enantiomer with a retention time of 3.94 min and 98.6% ee. The (R) enantiomer had a retention time of 3.55 min and 98.0% ee.

(S)-3-(Aminomethyl)-4-bromo-7-ethoxybenzo[c][1,2]oxaborol-1(3H)-ol hydrochloride (compound 14). A solution of *tert*-butyl (S)-((7-ethoxy-1-hydroxy-1,3-dihydrobenzo[c][1,2]oxaborol-3-yl)methyl)carbamate (8.0 g, 26.1 mmol), NBS (5.4 g, 30.6 mmol) and 2,2'-azobis(2-methylpropionitrile (500 mg) in CH_3CN (1500 mL) was stirred at 90 °C for 1 h. The reaction mixture was then concentrated *in vacuo*. The residue was purified by prep HPLC eluted with water/acetonitrile gradient (0.025% TFA) to give *tert*-butyl (S)-((4-bromo-7-ethoxy-1-hydroxy-1,3-dihydrobenzo[c][1,2]oxaborol-3-yl)methyl)carbamate as a

white solid (7.0 g, yield 69%). ^{1}H NMR (400 MHz, DMSO-d_6) δ 8.90 (s, 1H), 7.49-7.51 (d, 1H), 6.72-6.80 (d, 1H), 6.70 (m, 1H), 5.04-5.05 (d, 1H), 4.07-4.08 (m, 2H), 3.79-3.80 (d, 1H), 3.05-2.98 (m, 1H), 1.27-1.32 (m, 12H). A mixture of *tert*-butyl (S)-((4-bromo-7-ethoxy-1-hydroxy-1,3-dihydrobenzo[c][1,2]oxaborol-3-yl)methyl)carbamate (7.0 g, 18.1 mmol) in HCl/EtOAc (250 ml) was stirred for 2 h then concentrated *in vacuo*. The solid was washed with *n*-hexane and was then filtered, dried *in vacuo* to give (S)-3-(aminomethyl)-4-bromo-7-ethoxybenzo[c][1,2]oxaborol-1(3H)-ol hydrochloride (**compound 14**) as a white solid (5.30 g, yield 91%). ^{1}H NMR (400 MHz, DMSO-d_6) δ 9.14 (s, 1H), 8.39 (bs, 3H), 7.61-7.63 (d, 1H), 6.91-6.93 (d, 1H), 5.31-5.33 (d, 1H), 4.12-4.07 (q, 2H), 3.60-3.63 (d, 1H), 2.84-2.89 (m, 1H), 1.32-1.35 (t, 3H); ESI-MS m/z = 286 $[M + H]^+$; HPLC purity: 99.6% (220 nm). Analysis of its BOC-derivatized material using a Chiralpak AD-H analytical column showed the (S) enantiomer with a retention time of 1.46 min and 98.6% ee. The (R) enantiomer had a retention time of 1.30 min and 99.9% ee.

REFERENCES

1. **Cheng, A. F., W. W. Yew, E. W. Chan, M. L. Chin, M. M. Hui, and R. C. Chan.** 2004. Multiplex PCR amplimer conformation analysis for rapid detection of gyrA mutations in fluoroquinolone-resistant *Mycobacterium tuberculosis* clinical isolates. Antimicrobial agents and chemotherapy **48:**596-601.

2. **Hernandez, V., T. Crepin, A. Palencia, S. Cusack, T. Akama, S. J. Baker, W. Bu, L. Feng, Y. R. Freund, L. Liu, M. Meewan, M. Mohan, W. Mao, F. L. Rock, H. Sexton, A. Sheoran, Y. Zhang, Y. K. Zhang, Y. Zhou, J. A. Nieman, M. R. Anugula, M. Keramane el, K. Savariraj, D. S. Reddy, R. Sharma, R. Subedi, R. Singh, A. O'Leary, N. L. Simon, P. L. De Marsh, S. Mushtaq, M. Warner, D. M. Livermore, M. R. Alley, and J. J. Plattner.** 2013. Discovery of a novel class of boron-based antibacterials with activity against Gram-negative bacteria. Antimicrobial agents and chemotherapy **57:**1394-1403.

Table S1. Data collection and refinement statistics

M. tuberculosis LeuRS + AMP	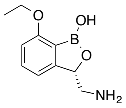 Compound 2	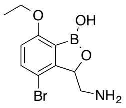 Compound 11	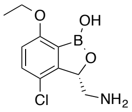 Compound 13
**Data collection**			
Space group	$P2_12_12_1$	$P2_12_12_1$	$P2_12_12_1$
Cell dimensions			
a, *b*, *c* (Å)	44.33, 61.85, 77.08	44.75, 61.16, 76.99	43.88, 61.88, 76.88
α, β, γ (°)	90.00, 90.00, 90.00	90.00, 90.00, 90.00	90.00, 90.00, 90.00
Resolution (Å)[a]	39-1.30 (1.30-1.34)	39-1.47 (1.47-1.51)	20-1.45 (1.45-1.49)
R_{sym}	4.6 (84.7)	5.3 (70.6)	4.4 (60.4)
$I / \sigma I$	14.4 (1.8)	17.1 (2.2)	17.9 (3.0)
Completeness (%)	96.7 (98.9)	99.3 (98.4)	98.3 (98.3)
Redundancy	4.3 (4.0)	4.3 (4.0)	3.9 (4.0)
**Refinement**			
Resolution (Å)	1.3	1.47	1.45
No. reflections work/free	48494/2668	35180/1857	35309/1841
R_{work} / R_{free}	0.171 / 0.197	0.183 / 0.206	0.183 / 0.210
No. atoms			
Protein	1504	1528	1485
Ligand	37 [2-AMP]	38 [Cmp 11-AMP]	38 [Cmp 13AMP]
Water/other	267	277	306/1 glycerol
B-factors			
Protein	14.8	13.9	17.7
Ligand	12.9	10.7	15.9
Water/other	37.6	30.2	35.8/glycerol 33.4
R.M.S. deviations			
Bond lengths (Å)	0.008	0.010	0.008
Bond angles (°)	1.51	1.56	1.62

[a] Values in parentheses are for highest-resolution shell.

Table S2.

***M. tuberculosis* H37Rv Single-step resistant mutants generated to compound 13.**

Strain	SNP	Agar MIC (µg/mL)
M. tuberculosis H37Rv	WT	0.37 – 0.75
RM1	*leuS* S311L	3.0
RM2	*leuS* S311L	3.0
RM3	*leuS* S311L	6.0
RM4	*leuS* S311L	6.0
RM5	*leuS* S311L	12
RM6*	*leuS* L432P	12
RM7*	*leuS* S311L	>24
RM8	*leuS* S311L	>24

RM = resistant mutant, *SNP derived from whole genome sequencing

Table S3. ***In vitro*** **activity against multidrug-resistant** ***M. tuberculosis*** **isolates**

Strain	Cmp13 (µg/mL)	Cmp14 (µg/mL)	Isoniazid (µg/mL)
M. tuberculosis H37Rv	0.015	0.030	0.060
M. tuberculosis M70	0.015	0.030	>0.12
M. tuberculosis M28	0.030	0.030	>0.12
M. tuberculosis M50	0.030	0.030	>0.12
M. tuberculosis TN5904	0.015	0.030	>0.12

The *M. tuberculosis* M70 strain was isolated in Hong Kong and is resistant to fluoroquinolones (FQ), streptomycin (STR), isoniazid (INH), rifampicin (RIF) and pyrazinamide (PZA). The *M. tuberculosis* M28 strain was isolated in Hong Kong and is resistant to FQ, INH, RIF, ethambutol and PZA. The *M. tuberculosis* M50 strain was isolated in Hong Kong and is resistant to FQ, STR, INH, RIF and PZA. The *M. tuberculosis* TN5904 strain was isolated in New Jersey, USA and is resistant to STR, INH, RIF and PZA (1).

FIG. S1

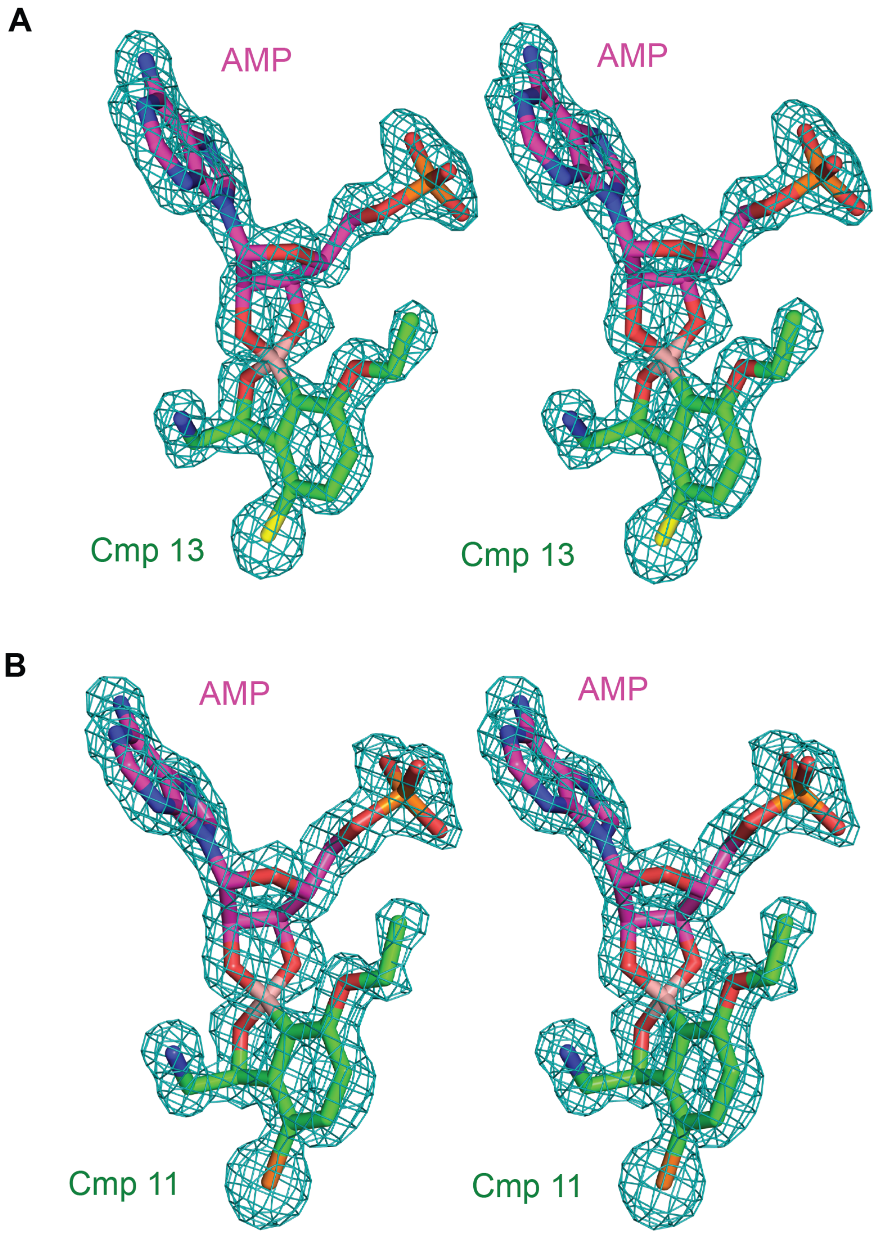

Supplementary Figure 1. Stereo diagram showing unbiased m(Fo-Fc) difference electron density (contoured at 3.5 σ) for the adducts formed by the compound 13 (top) and compound 11 (bottom) with AMP in the editing site of Mtb LeuRS.

FIG. S2

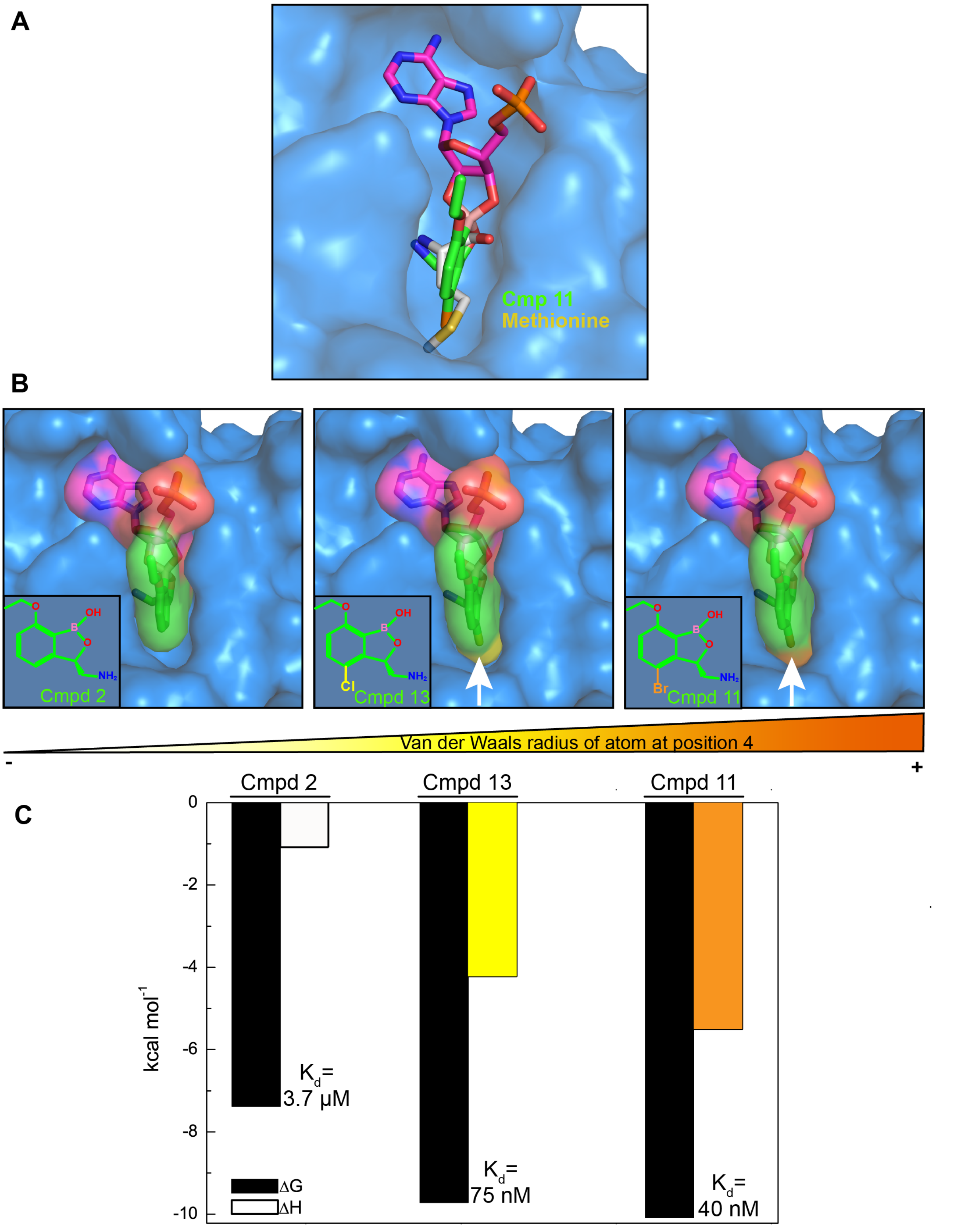

Supplementary Figure 2. Potency of compounds as a function of the size of the atom at position 4. (a) Overlay of the LeuRS editing domain of M. tuberculosis and E. coli in complex with methionine coloured in yellow (PDB: 2AJF). (b) Calculated Van der Waals surfaces for the editing domain of M. tuberculosis LeuRS in complex with the compounds 2 (left), 13 (middle) and 11 (right). The color code is the same as in Figure 1, except that the chlorine is represented in yellow and the bromine in orange. Arrow depicts the position occupied by the halogen atoms (c) Gibbs free energies (black bars) extracted from the calorimetric titrations of compounds 2, 13 and 11 into M. tuberculosis LeuRS. The binding enthalpies showing the contribution of 4-Cl and 4-Br compounds are shown as yellow and orange bars, respectively.
